# Patterns of Genomic Integration of Nuclear Chloroplast DNA Fragments in Plant Species

**DOI:** 10.1093/dnares/dst045

**Published:** 2013-10-29

**Authors:** Takanori Yoshida, Hazuka Y. Furihata, Akira Kawabe

**Affiliations:** Faculty of Life Sciences, Kyoto Sangyo University, Kyoto, Kyoto 603-8555, Japan

**Keywords:** NUPTs, chloroplast, nuclear plastid DNA, plant, evolution

## Abstract

The transfer of organelle DNA fragments to the nuclear genome is frequently observed in eukaryotes. These transfers are thought to play an important role in gene and genome evolution of eukaryotes. In plants, such transfers occur from plastid to nuclear [nuclear plastid DNAs (NUPTs)] and mitochondrial to nuclear (nuclear mitochondrial DNAs) genomes. The amount and genomic organization of organelle DNA fragments have been studied in model plant species, such as *Arabidopsis thaliana* and rice. At present, publicly available genomic data can be used to conduct such studies in non-model plants. In this study, we analysed the amount and genomic organization of NUPTs in 17 plant species for which genome sequences are available. The amount and distribution of NUPTs varied among the species. We also estimated the distribution of NUPTs according to the time of integration (relative age) by conducting sequence similarity analysis between NUPTs and the plastid genome. The age distributions suggested that the present genomic constitutions of NUPTs could be explained by the combination of the rapidly eliminated deleterious parts and few but constantly existing less deleterious parts.

## 1. Introduction

In general, two cytoplasmic organelles in plants have their own genomes: chloroplast and mitochondria. These organelles have been thought to have ac-quired these genomes via endosymbiotic mechanism during the early evolution of eukaryotic species.^1,2^ Chloroplasts are considered to have originated from cyanobacteria, although their genome size (around 150 kb) and gene number (about 100 protein-coding genes) are remarkably reduced compared with those of cyanobacteria (genome size, around 7 Mb; gene number: more than 5000 protein-coding genes).^3,4^ The major parts of the chloroplast genome were simply eliminated since they were redundant with the nuclear genes. Furthermore, some other parts of the cytoplasmic genome had been transferred to the nuclear genome, and the original ones were lost.^3,5^ The transfer of DNA fragments occurred between three genomes, although the direction of transfer was not uniform. Transfer from the cytoplasmic-to-nuclear genome is considerably higher than the opposite, and transfer between organelle genomes is considered to be rare.^6,7^ The pattern of chloroplast DNA integration in the nuclear genome (so-called nuclear plastid DNA: NUPT) has been investigated in several species, and the mechanism of their integration and genomic organizations has been analysed in detail.^7,8^

The transfer of DNA fragments from the chloroplast-to-nuclear genome is still active.^9–11^ The actual transfer rate was estimated to be about one per 16,000 pollen grains (about 6 × 10^–5^ per pollen grain)^9^ or one per 5 × 10^6^ cells (2 × 10^−7^ per cell)^11^ by measuring functional gene integration into the nuclear genome. When the length and functional ability of genes were considered, the total transfer rate of DNA fragments from the chloroplast-to-nuclear genome would be higher than that estimated by direct experiments.^9,11,12^ The transfer of chloroplast fragments occurred via both simple and complex structural organizations.^10^ These fragments form continuous, rearranged, inter-chromosomal rearranged, and mosaic structured patterns in the nuclear genome.^6^ NUPTs also tend to be located close to each other,^13^ suggesting simultaneous integration and/or biased integration preferences.^14^ Although the integration mechanism of *de novo* NUPTs is still not completely elucidated, non-homologous recombination and/or non-homologous end joining of double-strand break repair are suggested to be the integration mechanism as any other extra-nuclear genome DNAs.^15,16^ The integration mainly occurs during male gametogenesis^17^ and is increased by environmental stresses.^18,19^ After the chloroplast DNA fragments became integrated into the nuclear genome, newly formed NUPTs are sometimes unstable and are lost rapidly.^20^ Even though some NUPTs escape from the early unstable stage, they are fragmented and eliminated from the genome.^13,14,21^

Although the integration mechanisms, genomic organization, and evolution of NUPTs have been analysed in detail, most studies were performed using *Oryza sativa* and *Arabidopsis thaliana*.^3,14,21–23^ Despite frequent transfer and loss of genomic regions during evolution, the size and structure of the chloroplast genome is conserved among species, facilitating the elucidation of the general trend of DNA fragment transfer. Furthermore, because of its simple structure, chloroplast genome sequences have been determined from much more species, unlike mitochondrial genomes. In recent years, whole-genome sequences of >20 phylogenetically diverse plants have been published. Presently, estimating the genomic organization of NUPTs in these plant species has become possible, facilitating an understanding of the general rules of structural patterns and evolutionary history of NUPTs. Here, we report the amounts and structures of NUPTs from 17 plant species and discuss the general patterns that underlie the acquisition, maintenance, and elimination of nuclear-localized chloroplast DNA fragments.

## 2. Materials and Methods

### 2.1. Data used

Of the species whose whole-genome sequences have been reported, 7 with sequences for all 3 genomes (nuclear, chloroplast, and mitochondrial) and 10 with nuclear and chloroplast genome sequences were used in this study. Because there are some similarities between chloroplast and mitochondrial genomes, the former seven species were analysed in detail to estimate the pseudo-positive detection rate of NUPTs that were possibly originated from the mitochondrial genome. These seven species were as follows: *A. thaliana*,^24,25^ *Carica papaya*,^26^ *Vitis vinifera*,^27,28^ *Lotus japonicus*,^29,30^ *O. sativa*,^31,32^ *Sorghum bicolor*,^33,34^ and *Zea mays*.^35,36^ The species with the data for nuclear and chloroplast genomes were as follows: *Medicago truncatula*,^37^ *Glycine max*,^38,39^ *Manihot esculenta*,^40,41^ *Ricinus communis*,^42,43^ *Populus trichocarpa*,^44^ *Cucumis sativus*,^45,46^ *Fragaria vesca*,^47^ *Solanum lycopersicum*,^48,49^ *Solanum tuberosum*,^50,51^ and *Brachypodium distachyon*.^52,53^

### 2.2. Identification of NUPTs

The organelle genome sequences of each species were used as query to conduct BLAST search against nuclear genome sequences available in July 2012. In many cases, NCBI BLAST server was used; and bulk data downloaded from Phytozome ver 8.0^54^ or species-specific websites (*L. japonicus*, *F. vesca*, *S. tuberosum*, and *S. lycopersicum*) were also used to conduct local BLAST searches. The sequence dataset used in this study is listed in Supplementary Tables S1 and S2. In this study, only BLAST hits with 100 bp and longer in length and 90% and more identity to the chloroplast genome sequences were used for the following analyses. Because NUPTs were present as fragmented and mosaic structures, some sequences detected by BLAST search were very short. BLAST scores tend to be low for short-length homologous sequences; therefore, we used sequence identities as cut-off criteria. These criteria might have limited our analysis to only relatively recent transfer events.

BLAST hits for the NUPTs found within inverted repeat regions of the chloroplast genome were obtained in both inverted repeat regions and could not be distinguished. These BLAST hits were counted only once. Some BLAST hits were found at the edge of the inverted repeat region and were also present on the other side of the inverted repeat over a single copy region. These sequences were counted in the latter position only.

### 2.3. Identification of regions of NUPT origin on the chloroplast genome

The chloroplast genomic region from where NUPTs originated was identified by assigning each identified NUPT to a chloroplast sequence. If NUPTs existed within inverted repeat regions, they were simply counted as half. After BLAST hits were assigned, the number of NUPTs was counted on each nucleotide site of the chloroplast genome. The differences in the presence of NUPTs between inverted repeat regions and single copy regions were tested by the Welch two-sample *t*-test.

### 2.4. Estimation of age distribution

For each identified NUPT, the sequence identity to chloroplast genome was calculated. Although plant organelle genome is known to have a low- (1/10th) mutation rate compared with the nuclear genome,^55–57^ the exact nuclear/chloroplast mutation rate ratio is still unclear. Furthermore, because mutation directions differ between nucleotides in NUPTs,^58,59^ simple correction methods of genetic distances cannot be applied. Thus, *p*-distance was used to estimate the time of NUPT integration event (relative age). By using *p*-distances, the estimated age should represent relative age but not reflect true integration time. If natural selection affected evolution of the NUPTs, the substitution rate should vary especially if mutations were advantageous.

### 2.5. Characterization of flanking regions of NUPTs on the chromosomes

In the species analysed in this study, detailed information about transposons was obtained for *A. thaliana*, *O. sativa*, and *Z. mays* from Repbase.^60^ For these species, the number of transposable elements (TEs) in the vicinity of NUPTs was estimated using the program RepeatMasker ver.4.0.1 (available from: http://www.repeatmasker.org/) to investigate the features of the integrated regions of NUPTs. In all, 5-kb sequences from both 5′ and 3′ flanking regions of NUPTs were extracted. The number of each type of TE within the regions was estimated using RepeatMasker in the default mode. For comparison, the number of TEs within randomly extracted regions from genomes was estimated to compare with those found in the vicinities of NUPTs.

Some genomes retained NUPTs with substantial length and relatively low identities (i.e. predicted to be long-lived after insertion). In this study, NUPTs more than 5 kb in length with identities from 90 to 96% were considered as long-lived NUPTs. Flanking 5-kb regions were surveyed to analyse the surrounding region of long-lived NUPTs. Such long-lived NUPTs tended to be surrounded by other NUPTs or repeat sequences. First, the presence of other NUPTs surrounding long-lived NUPTs was verified. When long-lived NUPTs formed clusters with other NUPTs, the outer regions of such NUPT clusters were analysed as flanking regions of long-lived NUPTs. The occurrence of repeat sequences in the flanking region of long-lived NUPTs was analysed. Flanking 5-kb regions were used as query to conduct BLAST search against each nuclear genome sequence. If the entire or partial sequences in the 5-kb flanking regions were found >10 times in the whole genome, such sequences were tentatively defined as repeat sequences. The number of such repeat sequences in the flanking region was estimated.

## 3. Results and Discussion

### 3.1. Number and amount of NUPTs in plant species

The identified nuclear genome fragments similar to the chloroplast genome included several fragments that were also similar to the mitochondrial genome. Thus, we first estimated a proportion of sequences similar to both chloroplast and mitochondrial genomes (Table 1). Then, we estimated a proportion of NUPTs that are also similar to the mitochondrial genome. The results indicated that the values depended on the similarity between chloroplast and mitochondrial genome sequences. The proportion of indistinguishable sequences varied from 0.2 (*L. japonicus*) to 38.1% (*V. vinifera*) that were similar to that of sequences similar between chloroplast and mitochondrial genomes. The exceptions were *A. thaliana* and *O. sativa. Arabidopsis thaliana* had almost whole mitochondrial genome integration on Chromosome 2^61^ that caused inflation of the proportion of indistinguishable sequences. The reason for the low proportion of indistinguishable sequences in *O. sativa* was not clear. The results in the following analyses, however, were similar even if these indistinguishable fragments were included, suggesting similar evolutionary dynamics between NUPTs and nuclear mitochondrial DNAs (NUMTs) in a species. Thus, the ambiguous sequences were not excluded while performing the other analyses.

**Table 1.** Summary of homologous regions between chloroplast and mitochondria genomes

Species	Genome size (kb)		Homologous regions between chloroplast and mitochondria			NUPTs		
	Chloroplast	Mitochondria	Number	Length (bp)	Proportion to chloroplast genome (%)	Length (bp)	Also similar to mitochondrial genome	
							Length (bp)	Proportion (%)
*Arabidopsis thaliana*	154.5	366.9	8	3298	2.1	17 658	3167	17.9
*Carica papaya*	160.1	478.9	8	19 798	12.4	269 824	53 588	19.9
*Vitis vinifera*	160.9	773.3	36	63 033	39.2	337 711	128 698	38.1
*Lotus japonicus*	150.5	380.9	7	2687	1.8	147 286	239	0.2
*Oryza sativa*	134.6	490.5	15	24 770	18.4	846 607	42 326	5.0
*Sorghum bicolor*	140.8	468.6	16	24 328	17.3	169 352	27 013	16.0
*Zea mays*	140.4	569.6	10	21 729	15.5	1 006 782	160 965	15.5

The number and amount of NUPTs varied among species (Table 2). In all species, most identified NUPTs were short fragments (median ranged from 175 bp for *L. japonicus* to 514 bp for *P. trichocarpa*), and many of them are less than 200 bp (25% in *P. trichocarpa* to 65% in *L. japonicas*). *Arabidopsis thaliana* had only 38 NUPTs, and the total length of the NUPTs was about 18 kb. On the other hands, *Z. mays* had about 1500 NUPTs, and the total length was nearly 1 Mb. Previously, species with large genome sizes were shown to contain large amounts of NUPTs than those with small genome sizes.^62^ Similarly, in our study, a positive correlation existed between genome size and cumulative length of NUPTs (*P* < 0.01 by Kendall's rank correlation; Supplementary Fig. S1). However, the proportions of NUPTs in the nuclear genome were not constant among species. The lowest proportion was found in *M. esculenta*, which had only 10^−4^ of the nuclear genome showing similarity to its chloroplast genome. On the other hand, the monocot species *O. sativa* and dicot species *R. communis* had >0.2% nuclear genome similar to the chloroplast genome. The amount of NUPTs differed even between closely related species. In Gramineae species, the proportion of NUPTs varied from 0.024 (*S. bicolor*) to 0.222% (*O. sativa*). These differences might reflect differences in not only genome size but also genome complexity and proportion of repetitive elements and/or other factors. Because the assembly and annotation of the genome in most species has not yet been completed, additional NUPTs might be found in centromeres and chromosome knobs where the high amount of NUPTs would be expected.

**Table 2.** The amount of NUPTs in the plant species

Species	Genome size (Mb)^a^		NUPTs		Proportion to nuclear genome (%)
	Nuclear	Chloroplast	Number	Length (kb)	
*A. thaliana*	119	0.15	38 (31)	17.7 (14.5)	0.015 (0.012)
*C. papaya*	343	0.16	613 (486)	269.8 (216.2)	0.079 (0.063)
*V. vinifera*	486	0.16	900 (497)	337.7 (209.0)	0.069 (0.043)
*L. japonicus*	301	0.15	394 (392)	147.3 (147.0)	0.049 (0.049)
*M. truncatula*	567	0.12	361	477.8	0.084
*G. max*	974	0.15	1435	406.3	0.042
*M. esculenta*	533	0.16	199	54.4	0.010
*R. communis*	107	0.16	632	264.2	0.247
*P. trichocarpa*	481	0.16	293	241.8	0.050
*C. sativus*	203	0.15	169	49.0	0.054
*F. vesca*	195	0.16	218	58.2	0.030
*S. tuberosum*	727	0.16	563	429.6	0.059
*S. lycopersicum*	782	0.16	1513	674.4	0.084
*B. distachyon*	271	0.14	863	531.5	0.196
*O. sativa*	382	0.13	611 (495)	846.6 (804.3)	0.222 (0.210)
*S.bicolor*	697	0.14	515 (417)	169.4 (142.3)	0.024 (0.020)
*Z. mays*	2066	0.14	1459 (1099)	1041.3 (880.4)	0.050 (0.043)

In parentheses, the estimated values are shown when mitochondria–chloroplast transferred DNAs were excluded.

^a^Accumulative length of determined whole-genome sequences in database.

### 3.2. Distribution of NUPTs according to their origin from the chloroplast genome

**Figure 1.**
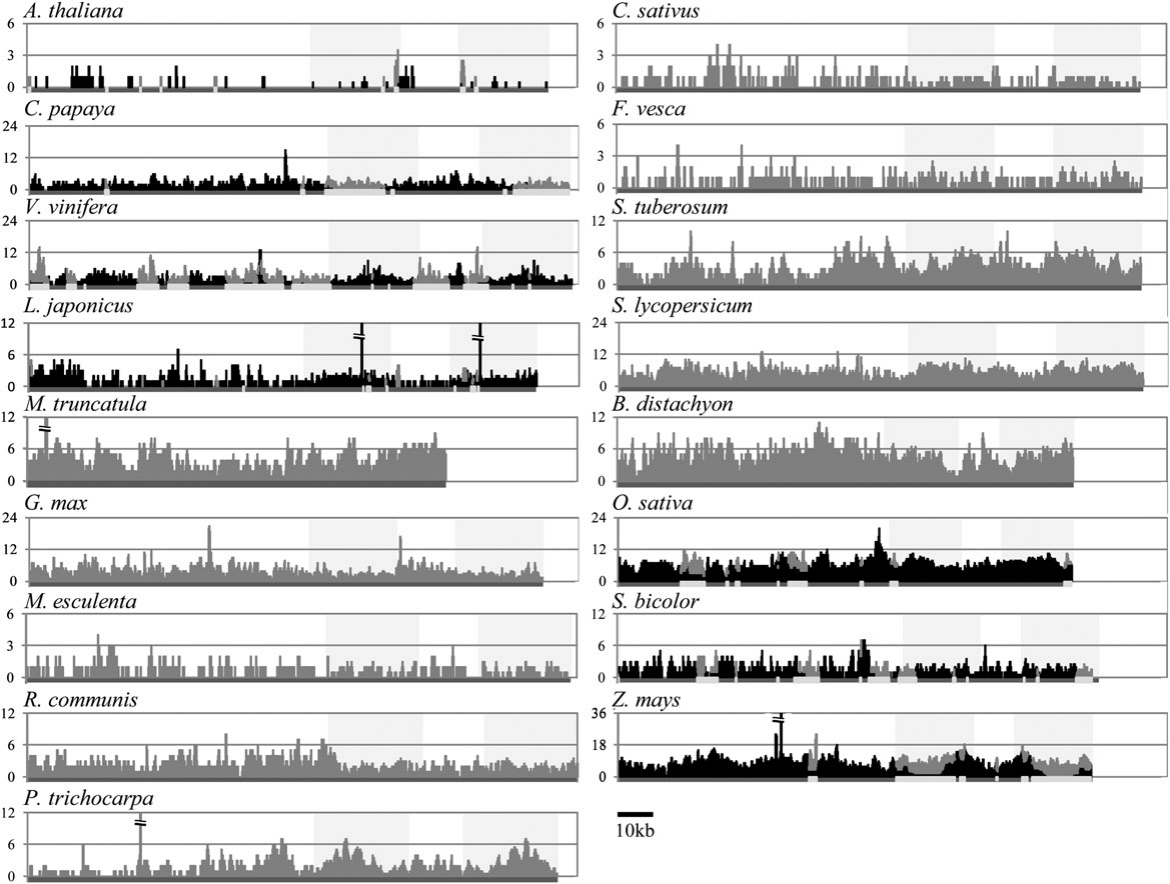
Distribution of NUPT sequences on the chloroplast genome. The amounts of NUPT origins on each chloroplast genome are shown for all 17 species. Numbers of NUPTs are plotted for each base pair of the chloroplast genome. For seven species with assembled mitochondrial genomes, NUPTs from the homologous regions between the chloroplast and mitochondrial genomes are shown by light gray bars, whereas other NUPT origins are shown by black bars. For the other 10 species, NUPT origins are shown by gray bars. Shaded boxes represent inverted repeat regions. Dark gray lines in the *x*-axis represent chloroplast genomes, where homologous regions between chloroplast and mitochondria are shown in white.

The structural differences among chloroplast genome regions could cause variation in the transfer rate to the nuclear genome. Thus, the incidence of transfer would not be uniform throughout the chloroplast genome. However, no regions had a extremely low or high amount of NUPTs in all species (Fig. 1), although there were significant differences in NUPT origin throughout the chloroplast genome. The absence of obvious hot or cold spots (regions) of NUPT origin suggests that no chloroplast genome regions have a strong deleterious effect on the host genome when transferred to the nuclear genome. Moreover, no resistance to transfer/integration mechanisms existed in the local chloroplast genomic regions. Taken together with the presence of long NUPTs, the fact that NUPTs frequently originated from not only genic regions but also intergenic regions of chloroplast genomes suggests that the integration predominantly occurred through DNA molecules as previously suggested.^63,64^

Matsuo *et al.*^21^ suggested that the high amount of NUPTs were present in the chloroplast–mitochondria homologous regions in rice. We also confirmed this biased origin in all species in which all three genome sequences were available (Supplementary Table S3). These results are reasonable because NUMTs cannot be distinguished from NUPTs in these regions, leading to the inflation of estimated NUPT numbers.

### 3.3. Biased distribution of NUPTs in the nuclear genome

**Figure 2.**
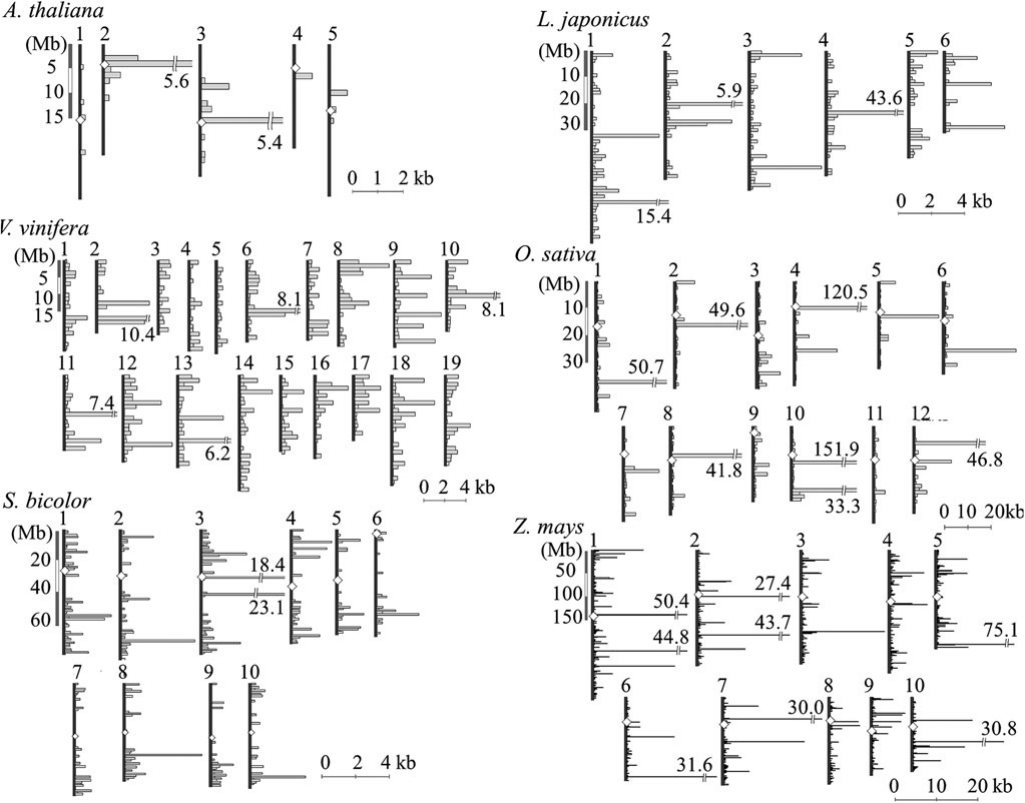
Position of NUPTs on the chromosomes. Locations and amounts of NUPTs are shown for six species (*A. thaliana*, *L. japonicus*, *V. vinifera*, *O. sativa*, *S. bicolor*, and *Z. mays*). Vertical lines and empty diamonds represent chromosomes and their centromeres. Nuclear genome was split into 1-Mb regions, and the amount of NUPTs in each region was plotted across a horizontal bar.

The amount of NUPTs also varied among nuclear chromosomal regions in each species (Fig. 2). Most species had regions with extremely large amount of NUPTs. In a 1-Mb region, the expected amount of NUPTs ranged from 100 bp to 2.5 kb in each species, but there were many regions that contained more than 5-kb NUPTs. Because NUPTs are known to be clustered in a specific region,^21,65^ several regions would be rich in NUPTs. NUPTs close to the centromere regions were suggested to be longer and younger in *O. sativa*.^21^ Michalovova *et al.*^66^ showed that the pericentromere-biased distribution of NUPTs was observed especially in species with a small genome size, whereas species with large genome sizes showed a wide distribution of NUPTs. We also observed that the distribution of NUPTs was not always similar among species (Fig. 2). In species in which centromere locations were known, high amount of NUPTs was detected not only in the regions close to the centromere, but also in the distal regions of the chromosomes, even though such distal regions often contain gene-rich regions. This finding might imply the difference in the tolerability of the amount of NUPTs in the distal regions across species.

**Figure 3.**
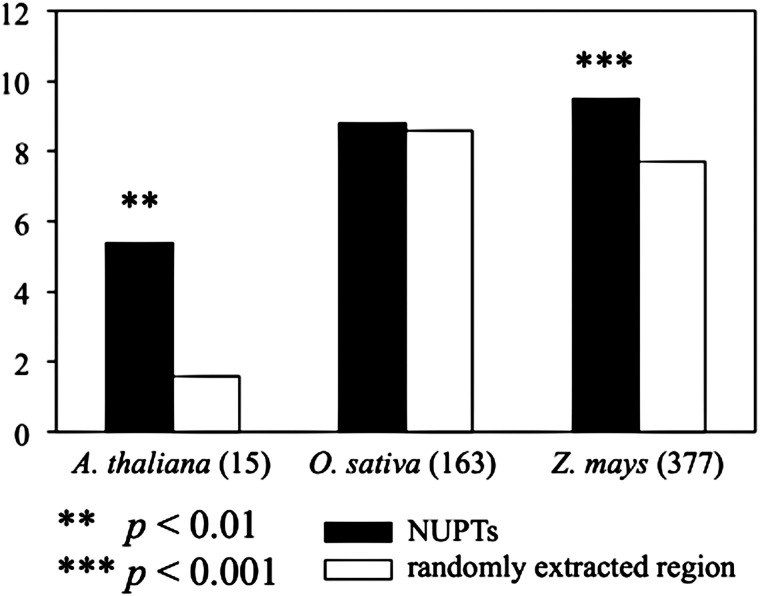
Types of TEs in the vicinity of NUPTs. The average number of TEs in the vicinity of NUPTs is shown. The 5-kb sequences from both 5′ and 3′ flanking regions were extracted to analyse the number of TEs. TEs in sequences were estimated using the program RepeatMasker (available from: http://repeatmasker.org). For comparison, the average number of TEs within randomly extracted regions from each genome was also estimated. The figures in parenthesis show the number of NUPTs analysed (with identity from 96 to 90%, ≥100 bp).

The high amount of NUPTs in specific chromosomal regions could be related to the presence of TEs. Because NUPTs are non-functional elements like TEs, the pressures of their degradation and elimination would be high in gene-rich regions, where foreign DNA insertions cause disruption and/or interruption of accurate gene function and regulation. Actually, the regions surrounding NUPTs were rich in TEs in all the three analysed species (Fig. 3). *Arabidopsis* *thaliana* and *Z. mays*, but not *O. sativa*, had significantly a higher amount of TEs around NUPTs, suggesting co-localization or similar genomic organization of NUPTs and TEs. Our findings imply the similarities between NUPTs and TEs that are considered as ‘junk’ DNA and show restricted distributions in the host genomes.

### 3.4. Age distribution of NUPTs

**Figure 4.**
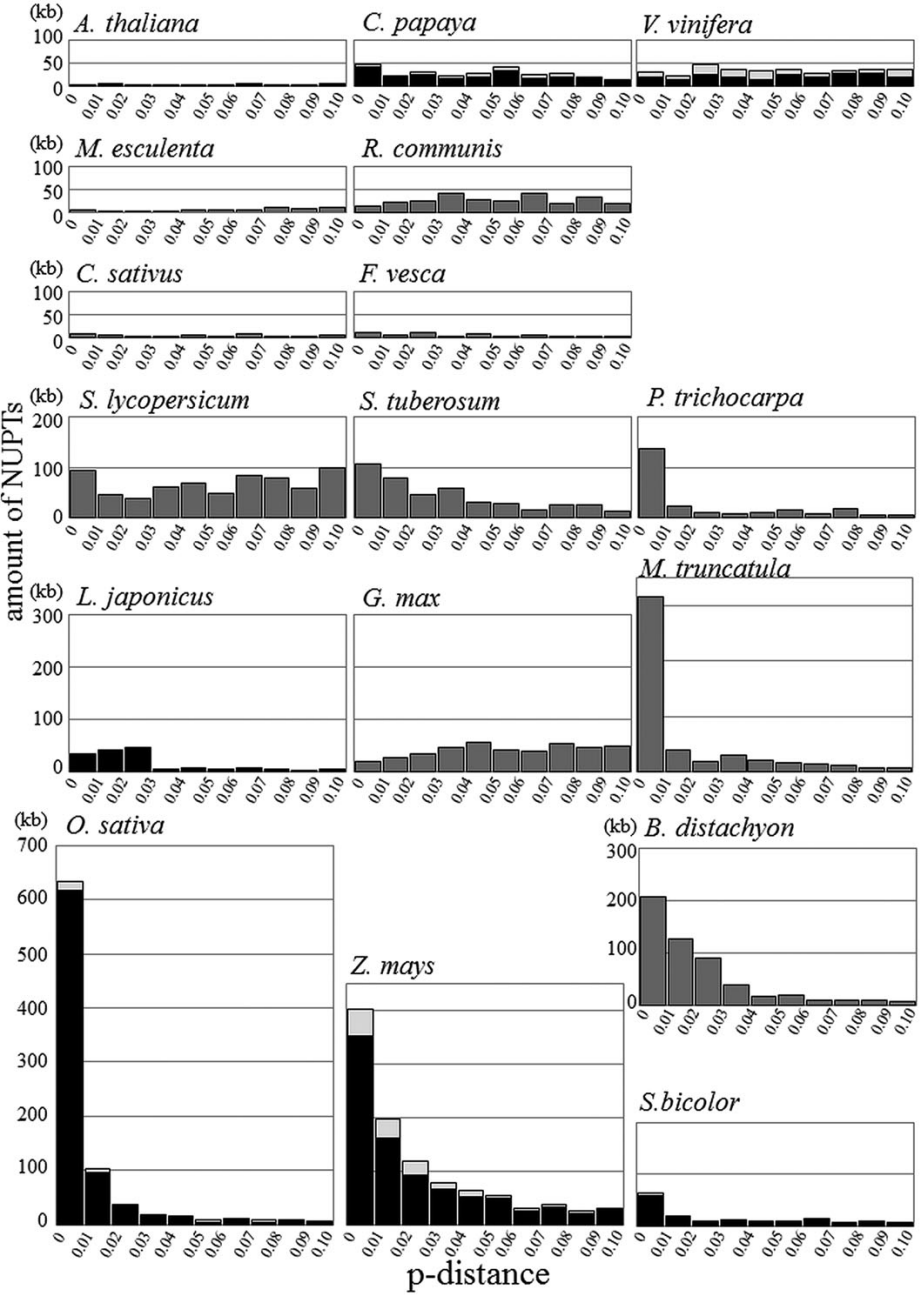
Age distribution of NUPTs. The amount of NUPTs for each 1% *p*-distance interval is shown for all 17 species. For seven species with assembled mitochondrial genomes, NUPTs from the homologous regions between chloroplast and mitochondrial genomes are shown by light gray bars, whereas other NUPT origins are shown by black bars. For the other 10 species, NUPT origins are shown by gray bars. The scale of the amount of NUPTs is the same in all species.

There were two distinct patterns of NUPT age distribution in the analysed plant species (Supplementary Fig. S2). One was typical for *A. thaliana* and most other dicot species, where no clear biases were observed. The other was found in all Gramineae species and few dicot species, showing very high proportion of young fragments that decreased dramatically with time. Although these two patterns were completely different, closely related species occasionally showed different patterns from each other. In Fabaceae, *G. max* had no biased age distribution of NUPTs, whereas *M. truncatula* showed a high peak at the youngest age. Although age distribution patterns differed between species, when the same scale of NUPT amount was used (Fig. 4), the two typical patterns could be explained by the combination of two different categories: the exponentially reduced part and a low constantly existing part. The distributions of Gramineae species and few dicot species (*P. trichocarpa* and *M. truncatula*) seem to be composed of these two categories, whereas those of other dicots would have a small portion of exponentially reduced part. These two categories might represent the difference of selective pressure against NUPTs in each category. We discussed this hypothesis later.

**Figure 5.**
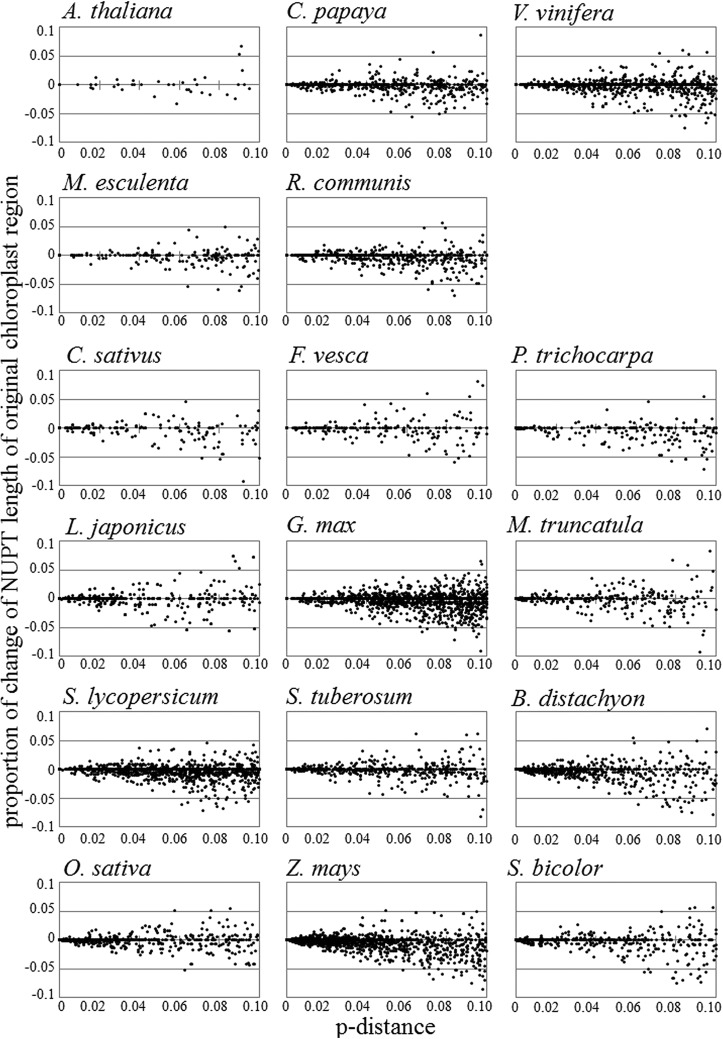
Pervasive change in NUPT length. The proportion of NUPT length changes to the corresponding chloroplast sequence length was plotted for individual NUPTs. Horizontal lines represent *p*-distance between NUPTs and chloroplast sequences. Vertical lines represent the proportion of the length change between NUPTs and chloroplast sequences.

In addition to the elimination of individual NUPTs, their length pervasively changed from that found in the original chloroplast regions (Fig. 5). Differences in NUPT length correlated with NUPT age, indicating time-dependent accumulation of indel variations. The cumulative length differences in age classes were mostly negative, suggesting NUPT lengths became shorter after integration into the nuclear genome (Supplementary Fig. S3). These results suggest that the degradation of NUPTs occurred by not only complete elimination of individual NUPTs, but also by small indel variations.

**Figure 6.**
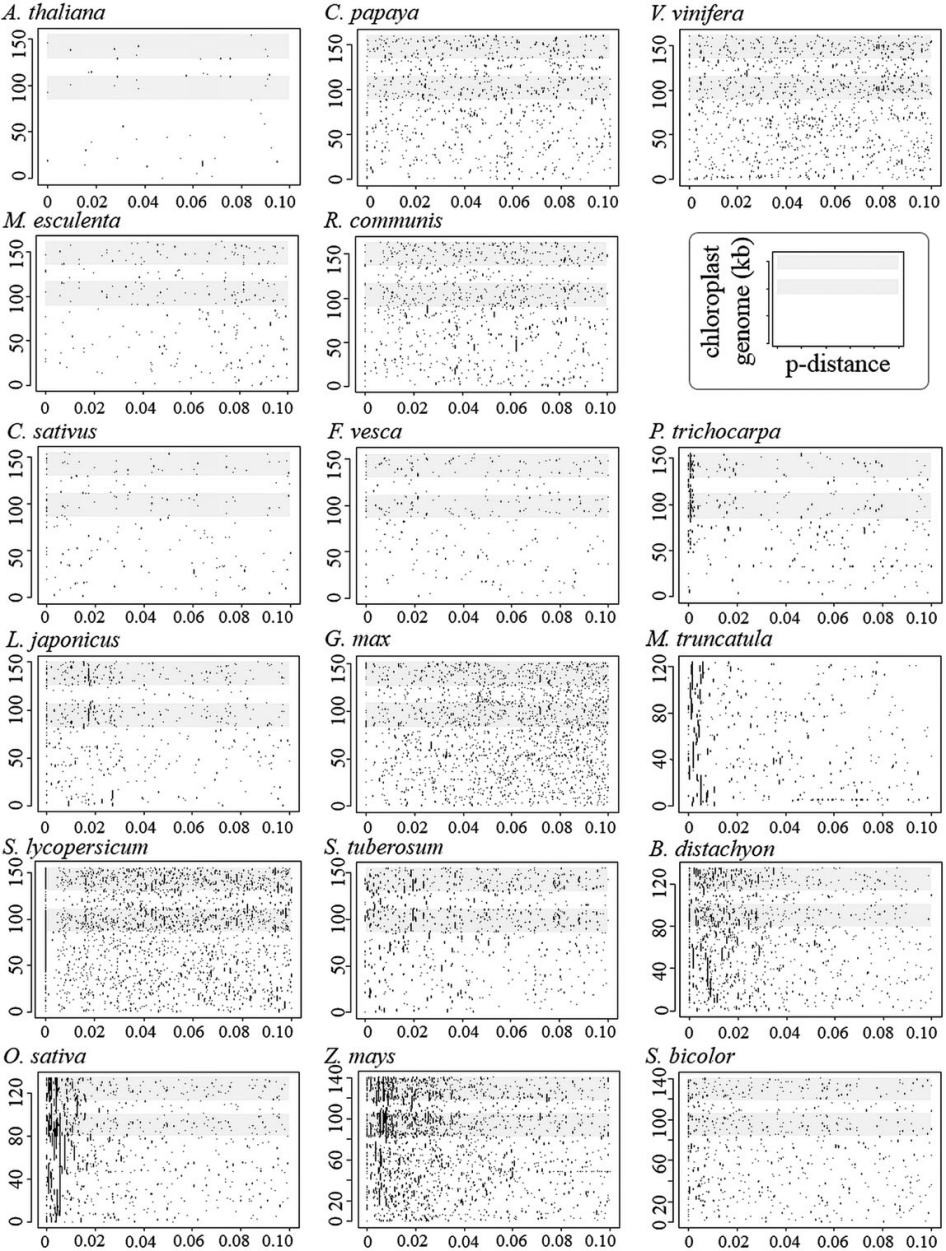
Relationship between age and length of NUPTs. Locations of NUPTs on the chloroplast genome are shown against *p*-distance for all 17 species. The shaded boxes represent inverted repeat regions.

Several NUPTs are known to be located in the same region with fragmentations and inversions.^14,21,65^ This phenomenon could have originated during the insertion process and create short-fragmented NUPTs around long NUPTs. Long NUPTs might have been degraded to short fragments or eliminated from the genome. This pattern was observed in many analysed species, especially in the species having numerous young-aged NUPTs (Fig. 6). In such species, some NUPTs were long as several tens of kb in size, but most of them had <4% *p*-distances to the chloroplast genome. This result suggests that the effective degradation and elimination occurred in all species, and that only small NUPTs can escape eliminations. It is of interest to know why some relatively long NUPTs (>5 kb) still exist without degradations. Such long-lived NUPTs might have been assigned new functions as genes or regulatory elements to be maintained under negative selection. The old NUPTs (>4% *p*-distances to the chloroplast genome) rarely maintained their lengths longer than 5 kb. Such long, old NUPTs were surrounded by short NUPTs (Table 3). Moreover, a high amount of repetitive sequences were located around the cluster of such NUPTs. These observations suggest that the long-lived NUPTs are relic of large NUPTs degraded to become fragmented, but not became functionary important.

**Table 3.** Number of long-lived NUPTs and characteristics of their flanking regions

Species	Number of long-lived NUPTs	Number of repeat sequences within 5-kb flanking regions
*A. thaliana*	1 (1)	1/10 kb
*C. papaya*	1 (0)	4/10 kb
*M. truncatula*	2 (0)	8/20 kb
*G. max*	1 (1)	6/10 kb
*R. communis*	1 (1)	5/10 kb
*P. trichocarpa*	1 (0)	more than 10/10 kb
*S. lycopersicum*	10 (10)	more than 10/70 kb
*Z. mays*	2 (2)	more than 10/20 kb

Numbers of NUPTs >5 kb in length and with a relatively low identity value (96–90%) are shown.

In the parentheses, the numbers of long-lived NUPTs having other NUPTs within 5 kb surrounding regions are shown.

### 3.5. Patterns of maintenance and elimination of NUPTs during evolution

The presence of NUPTs in the nuclear genome should be determined by the balance between the transfer and elimination rates during evolution. The transfer rate from the chloroplast-to-nuclear genome was estimated to be about 6 × 10^−5^ per pollen grain^9^ or 2 × 10^−7^ per cell^11^ by a direct experiment involving transgenic tobacco. These estimated values by direct experiments should be underestimate for the chloroplast genome transfer rate of any regions,^9,11,12^ because, in these experiments, antibiotic-resistant genes of about 1–1.5 kb were used and whole genic region, including promoter sequences, should be integrated in any chromosomal region where the integrated genes can become actively transcribed. Indeed, actual integrated DNA fragments were 6.0 to over 22.3 kb in length,^10^ suggesting chloroplast genome fragment integrations occurred by long DNA fragments. Such long-length NUPTs were very rare even in recently integrated sequences (0–6%; Supplementary Table S4). Although the direct experiment studies were carried by using tobacco alone, the actual transfer rate is valuable to consider the dynamics of NUPTs in plant genomes. By applying the values obtained in this study, we could roughly estimate the transfer rate of NUPTs in each plant species used in this study. Considering that all NUPTs are selectively neutral and using estimated transfer rate per generation, we found that the estimated number of NUPTs with <1% *p*-distances varied from 3 × 10^4^ (*O. sativa*) to 2 × 10^6^ (*V. vinifera*) (assuming neutral mutation rate per generation as 10^−8^). Even if the proportion of NUPTs longer than 1 kb was used for estimation, the values exceed over 3 × 10^4^. Each estimated value was at least 100-times larger than the observed number of NUPTs with <1% *p*-distances in each plant genome (Supplementary Table S4). This might be partly because of the overestimation of the NUPT amount to be <5 kb (or 1 kb). The number of shorter NUPTs could become greater by degradation than that of actually transferred fragments, although the effect might be limited. Rather, the difference between expected and observed NUPT numbers indicates high elimination pressure against the integration of NUPTs in all plant species. In the early evolutionary stage of endosymbiosis, the transfer of chloroplast fragments, especially including genes, would contribute an essential role for the tight regulation of chloroplasts by the host plant. However, recently transferred chloroplast genome fragments (young NUPTs) might have no clear functional roles and most of them were inactive.^67,68^ The rarity of functionally important NUPTs indicates that they resemble non-functional junk DNA, such as TEs. The NUPT integrations into functional genes or gene regulatory regions could cause severe deleterious effects. If NUPTs are non-functional and have deleterious effect, they can survive only in the regions where functional genes are rare. The large amount of long NUPTs close to the centromere region is reasonable since the elimination pressure would be less.

**Figure 7.**
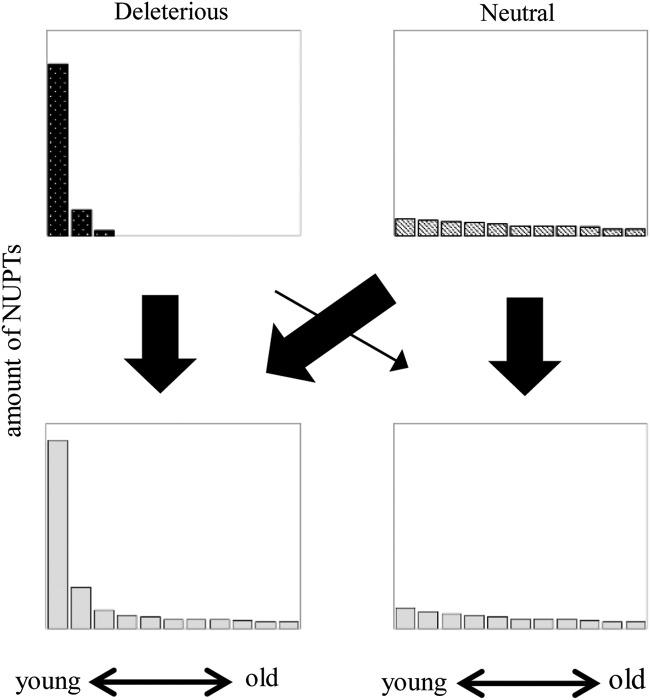
Schematic age distribution of NUPTs. Hypothetical age distributions of two different categories of NUPTs are shown. The left figure represents the distribution of deleterious insertions, whereas the right figure represents the distribution of insertions with no or few deleterious effects. The thickness of arrows indicates relative contribution of deleterious and neutral portion to two different categories of NUPTs distributions.

In this study, we found that the age distribution of NUPTs could be explained by the combination of the exponentially reduced part and uniformly existing part (Fig. 7). The former would be deleterious fragments and can survive in the gene-poor and TE-rich regions where eliminations are not effective. The uniformly existing parts are rare and short in all plant species. These old and short NUPTs possibly have less deleterious effect and have been maintained under neutrality. The different patterns of NUPT age distribution among plant species might reflect the amount of deleterious NUPTs in each species, or different integration rates among species that account for the amount of recently formed NUPTs. The pattern of degradation and elimination of integrated NUPTs is important especially for understanding the dynamics of exponentially reduced parts. Michalovova *et al.*^66^ suggested an important role of TEs on the mechanism of degradation and elimination of NUPTs. However, our results showed that there are many pervasive changes (small insertions and deletions within or including NUPTs) found in all species, indicating the relative importance of such indel variations for the degradation and elimination of NUPTs. Experimental studies using Tobacco suggest that rearrangements by pervasive changes occurred just after integration.^20,67^ Concomitant integration of multiple fragments could also form adjacent NUPTs from small disjunct parts of chloroplast genomes. Lloyd and Timmis^68^ analysed the patterns of integration and changes of *de novo* insertion of chloroplast DNA fragments to demonstrate integration by non-homologous end joining involving simultaneous insertion of several chloroplast DNA fragments from different chloroplast regions. The result suggested concomitant integration of three DNA fragments from different regions of the chloroplast genome. Although the relative importance of each factor that determine the pattern and dynamics of NUPTs is still unclear, both pervasive changes after integration and *de novo* concomitant integrations indicate the importance of early stage changes in the evolution of NUPTs. Detailed analyses on the elimination and degradation patterns of young NUPTs in different species might lead to the elucidation of the factor determining the amount of NUPTs in a species.

## Supplementary Data

Supplementary data are available at www.dnaresearch.oxfordjournals.org.

## Funding

This study was supported in part by Private University Strategic Research Foundation Support Program and grants-in-aid for Scientific Research in Innovative Areas (23125513 and 23113003) to A.K.

## Supplementary Material

Supplementary Data
