# Supplementary material for: Patterns of Genomic Integration of Nuclear Chloroplast DNA Fragments in Plant Species
Source: DNA Res. 2013 Oct 29;21(2):127–40. doi: 10.1093/dnares/dst045 (PMC3989485; doi:10.1093/dnares/dst045)
Supplement: Supplementary Data [file supp_21_2_127__index.html]

Patterns of Genomic Integration of Nuclear Chloroplast DNA Fragments in Plant Species — Patterns of Genomic Integration of Nuclear Chloroplast DNA Fragments in Plant Species — Supplementary Data 

# Patterns of Genomic Integration of Nuclear Chloroplast DNA Fragments in Plant Species

## Supplementary Data

Supplementary Data

**Files in this Data Supplement:**

- Supplementary Data - Doc file
- Supplementary Figure 1 - ppt file
- Supplementary Figure 2 - ppt file
- Supplementary Figure 3 - ppt file
- Supplementary Tables - doc file
