# Supplementary material for: Patterns of Genomic Integration of Nuclear Chloroplast DNA Fragments in Plant Species
Source: DNA Res. 2013 Oct 29;21(2):127–40. doi: 10.1093/dnares/dst045 (PMC3989485; doi:10.1093/dnares/dst045)
Supplement: Supplementary Data [file supp_dst045_dst045supp.doc]

Supplemental Figure 1. Relationship between genome size and total amount of NUPTs.

The correlation between nuclear genome size (Mb) and total amount of NUPTs (kb) is shown. The detailed values are shown in Table 2.

Supplemental Figure 2. Age distribution of NUPTs amount.

For convenience, each figure in Figure4 was re-scaled.

Supplemental Figure 3. Pervasive changes in NUPTs length.

The average proportion of NUPT length changes to the corresponding chloroplast sequence length was plotted for each 1% p-distance interval. The horizontal line represents p-distance. The vertical line represents the proportion of the length change between NUPTs and chloroplast sequences.
