# Supplementary figures and images for: Patterns of Genomic Integration of Nuclear Chloroplast DNA Fragments in Plant Species
Source: DNA Res. 2013 Oct 29;21(2):127–40. doi: 10.1093/dnares/dst045 (PMC3989485; doi:10.1093/dnares/dst045)

## Slide 1
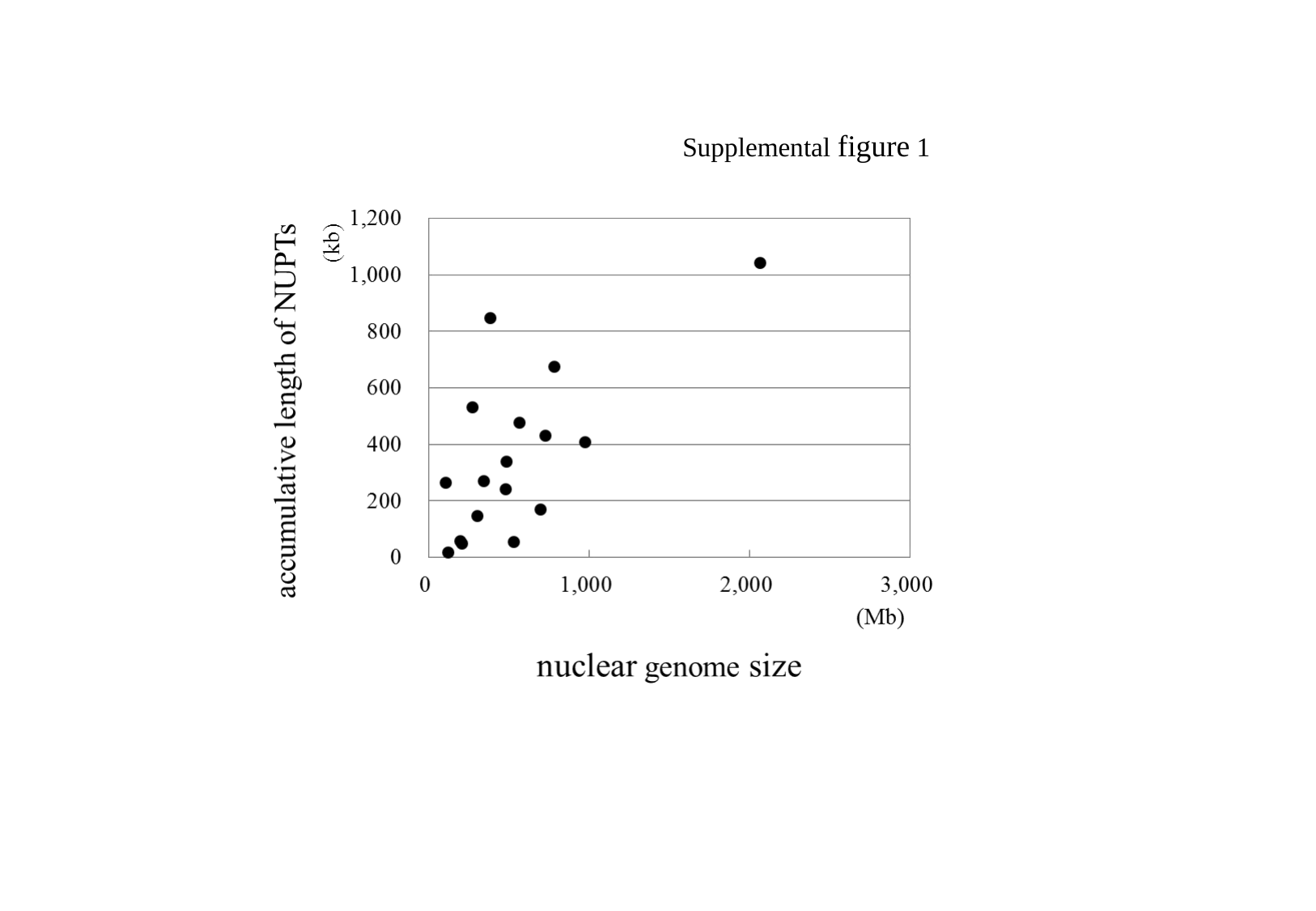

Supplemental figure 1

Supplement: Supplementary Data [file supp_dst045_dst045supp_fig1.ppt]
