# Supplementary material for: Patterns of Genomic Integration of Nuclear Chloroplast DNA Fragments in Plant Species
Source: DNA Res. 2013 Oct 29;21(2):127–40. doi: 10.1093/dnares/dst045 (PMC3989485; doi:10.1093/dnares/dst045)
Supplement: Supplementary Data [file supp_dst045_dst045supp_fig2.ppt]

## Slide 1
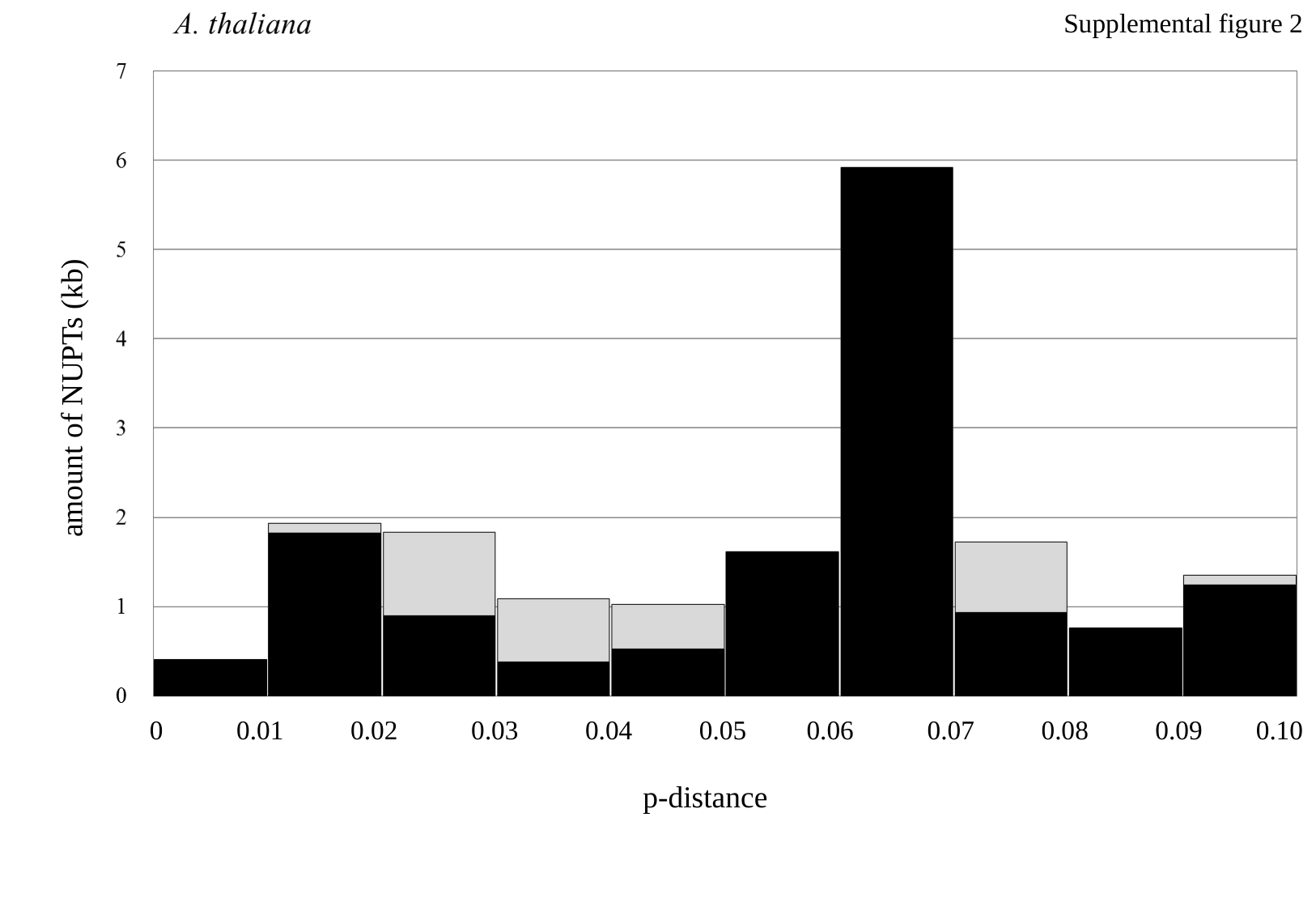

Supplemental figure 2
amount of NUPTs (kb)
 0 0.01 0.02 0.03 0.04 0.05 0.06 0.07 0.08 0.09 0.10
p-distance

## Slide 2
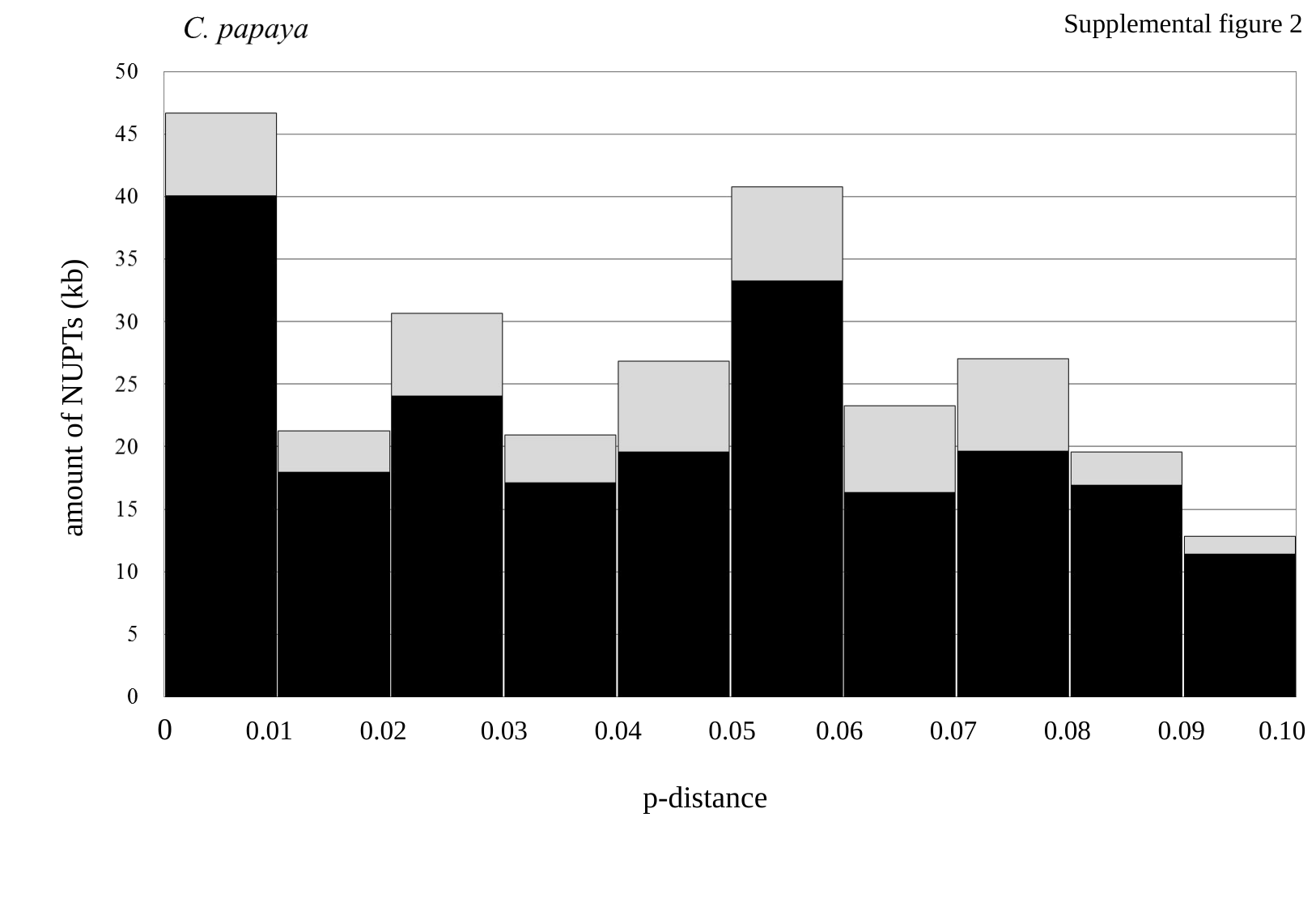

Supplemental figure 2
amount of NUPTs (kb)
 0 0.01 0.02 0.03 0.04 0.05 0.06 0.07 0.08 0.09 0.10
p-distance

## Slide 3
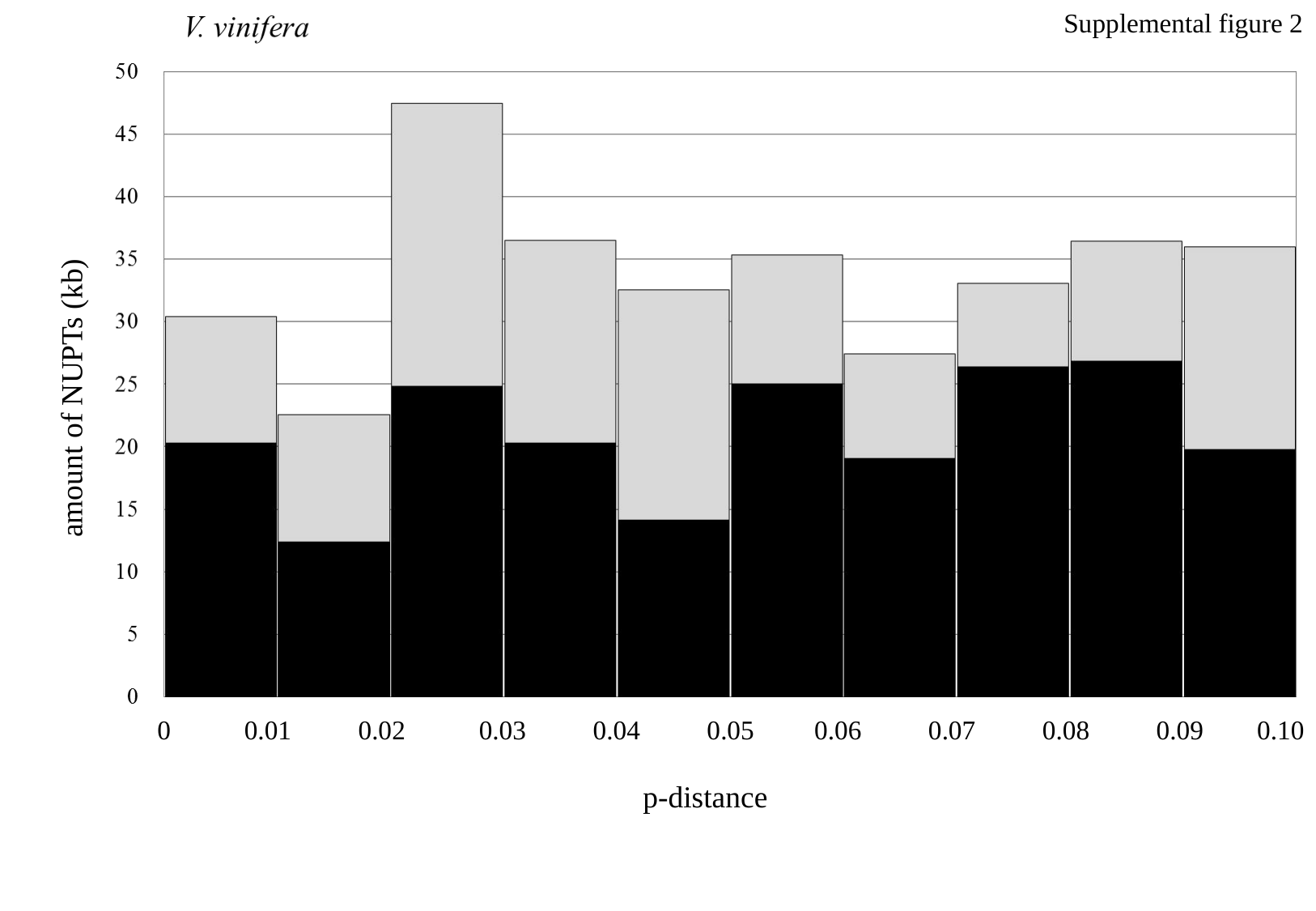

Supplemental figure 2
amount of NUPTs (kb)
 0 0.01 0.02 0.03 0.04 0.05 0.06 0.07 0.08 0.09 0.10
p-distance

## Slide 4
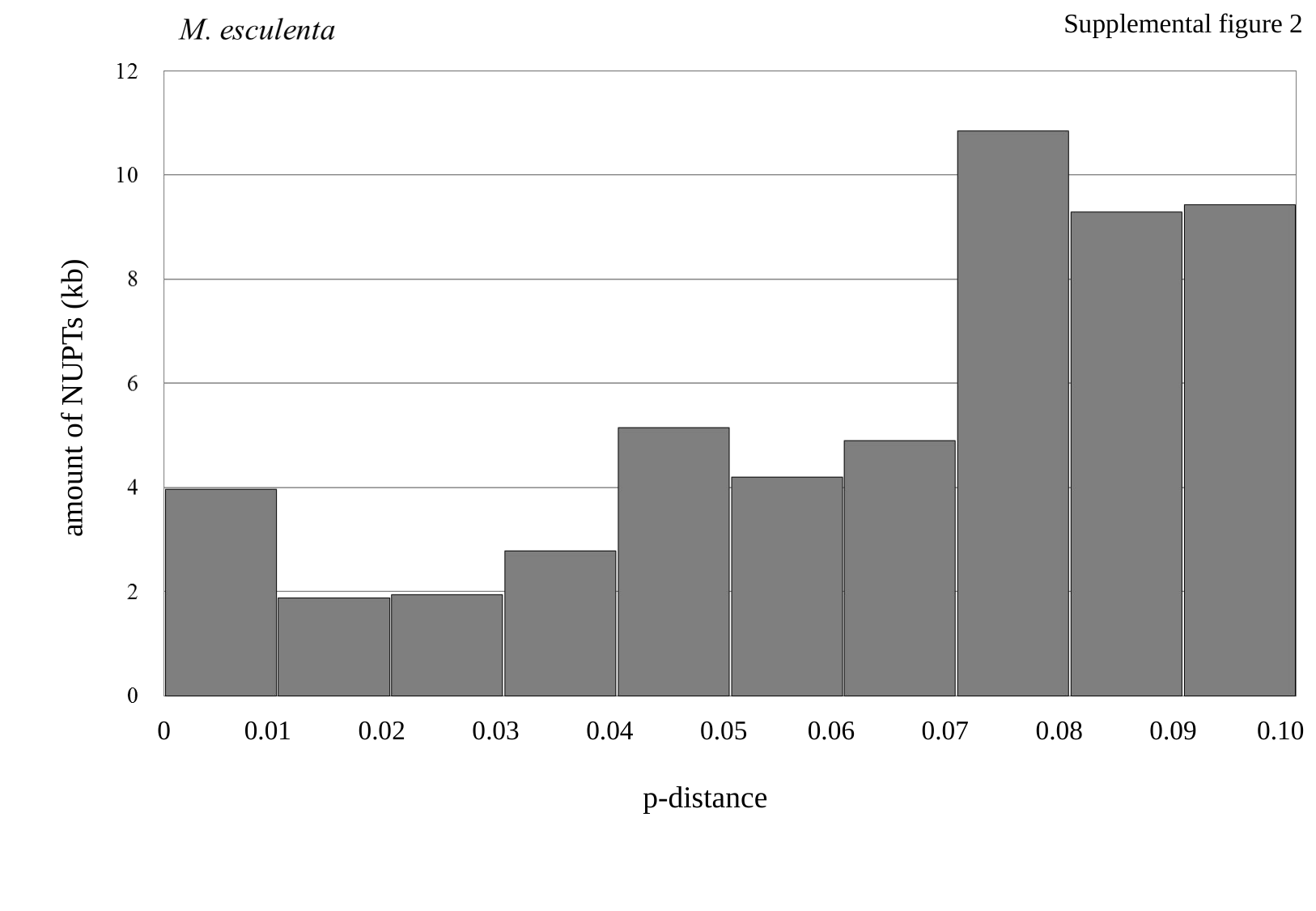

Supplemental figure 2
amount of NUPTs (kb)
 0 0.01 0.02 0.03 0.04 0.05 0.06 0.07 0.08 0.09 0.10
p-distance

## Slide 5
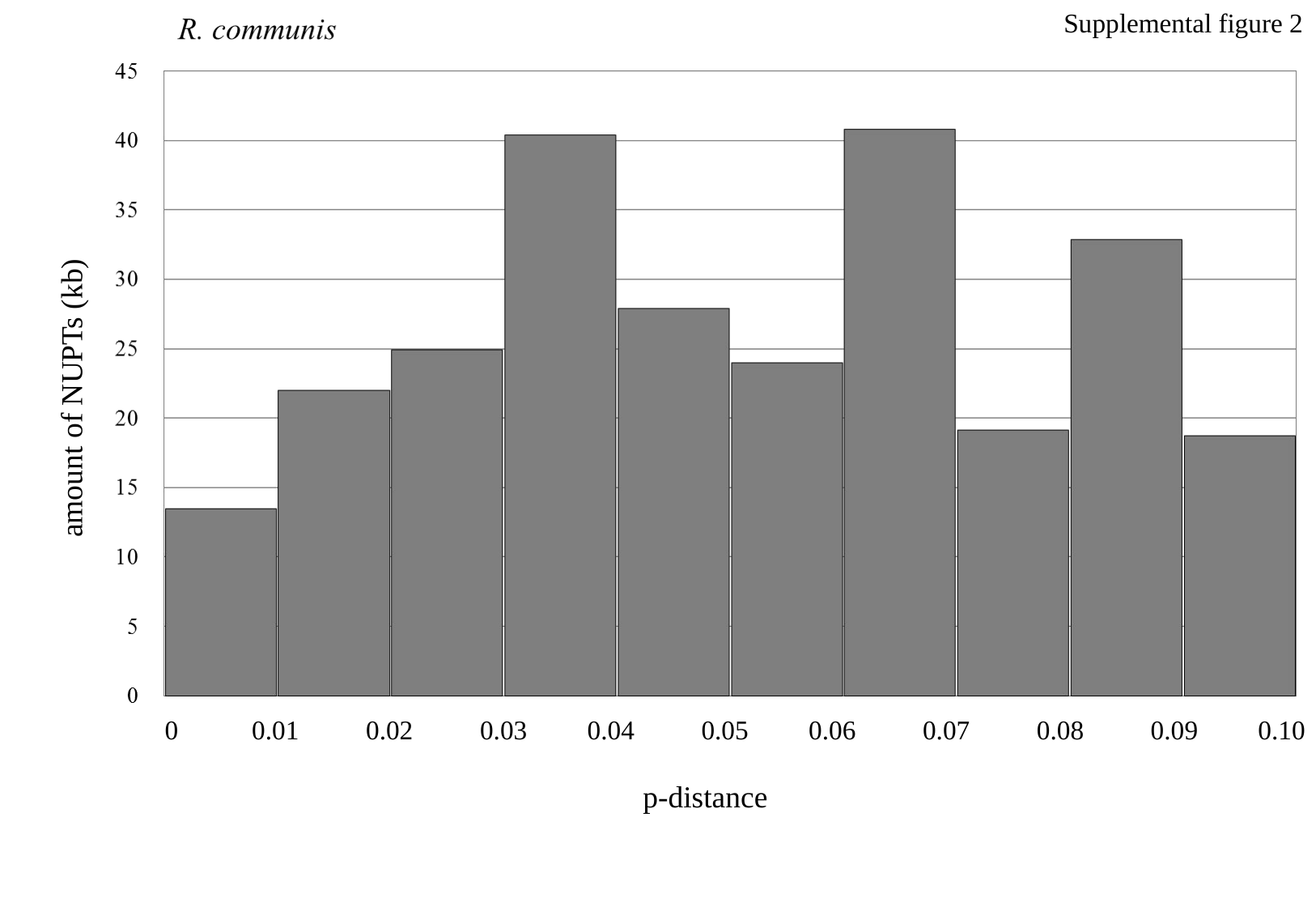

Supplemental figure 2
amount of NUPTs (kb)
 0 0.01 0.02 0.03 0.04 0.05 0.06 0.07 0.08 0.09 0.10
p-distance

## Slide 6
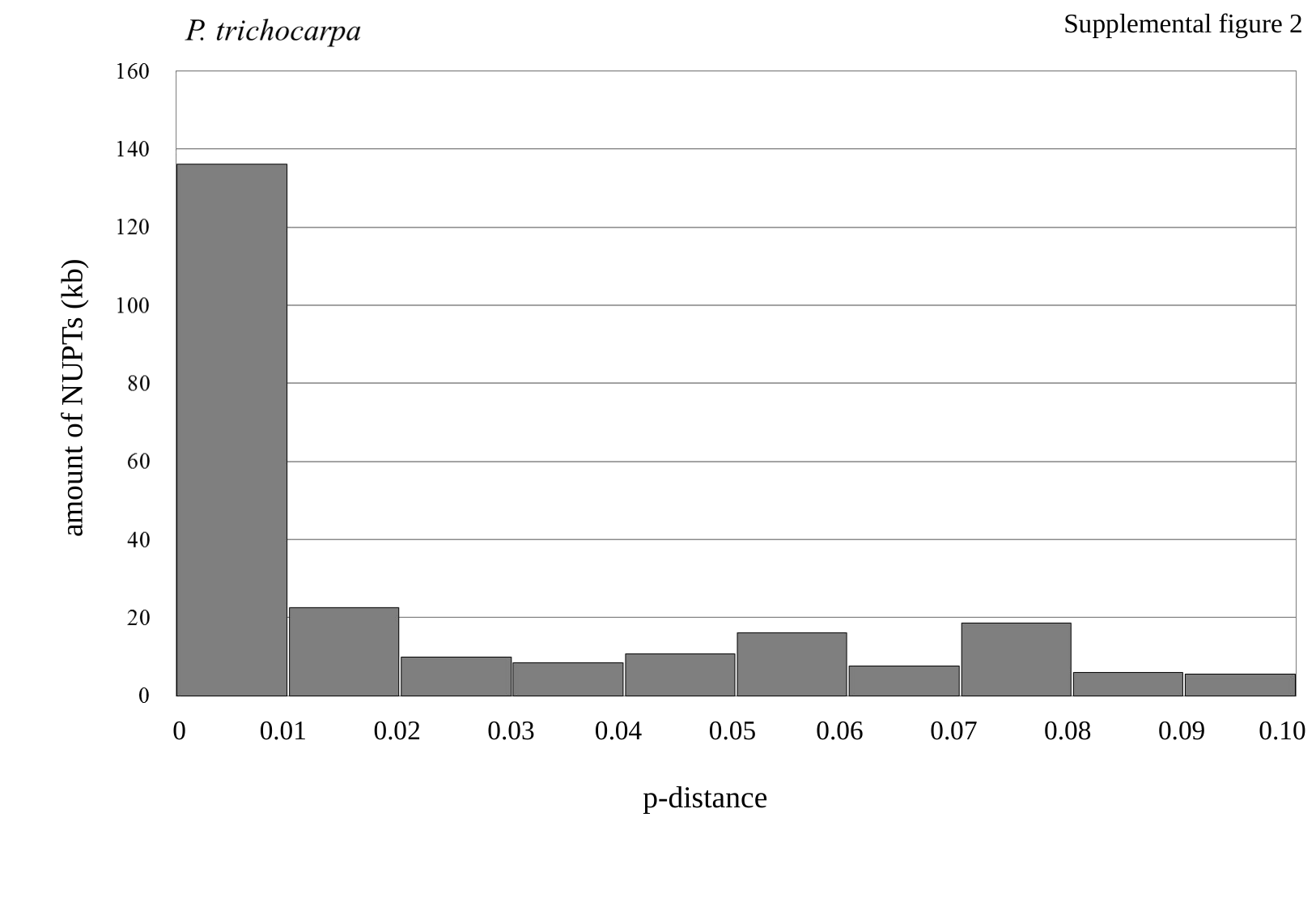

Supplemental figure 2
amount of NUPTs (kb)
 0 0.01 0.02 0.03 0.04 0.05 0.06 0.07 0.08 0.09 0.10
p-distance

## Slide 7
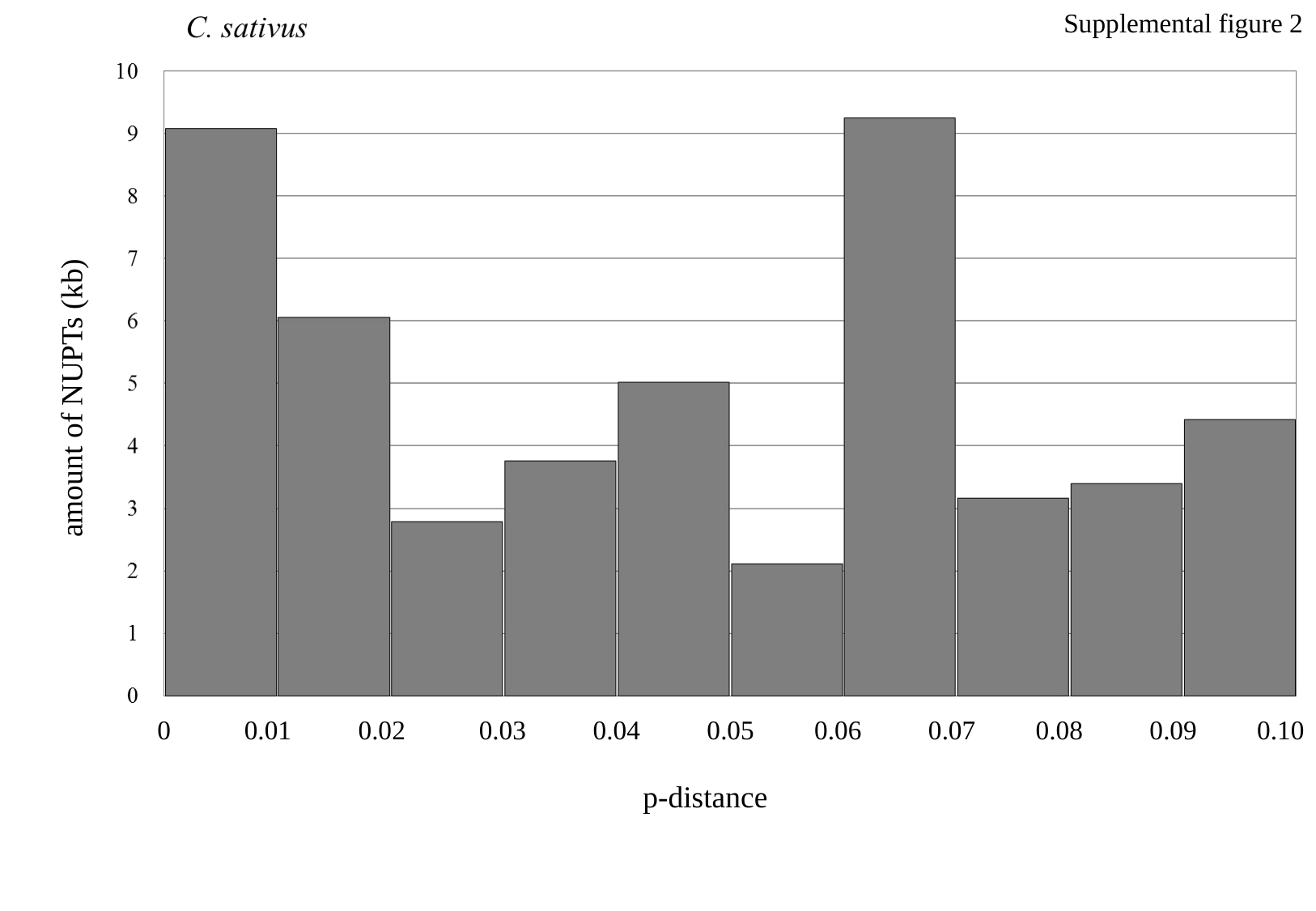

Supplemental figure 2
amount of NUPTs (kb)
 0 0.01 0.02 0.03 0.04 0.05 0.06 0.07 0.08 0.09 0.10
p-distance

## Slide 8
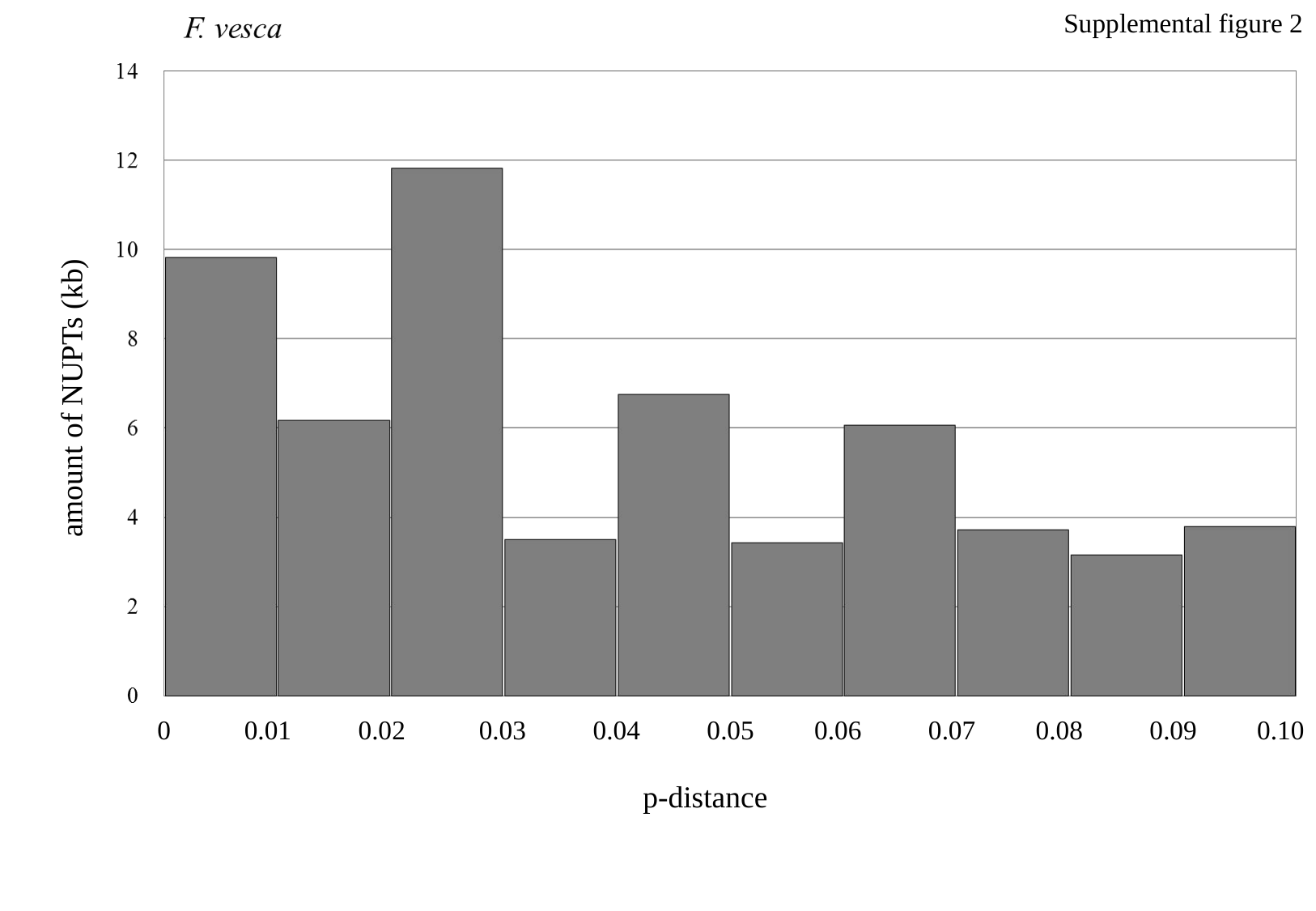

Supplemental figure 2
amount of NUPTs (kb)
 0 0.01 0.02 0.03 0.04 0.05 0.06 0.07 0.08 0.09 0.10
p-distance

## Slide 9
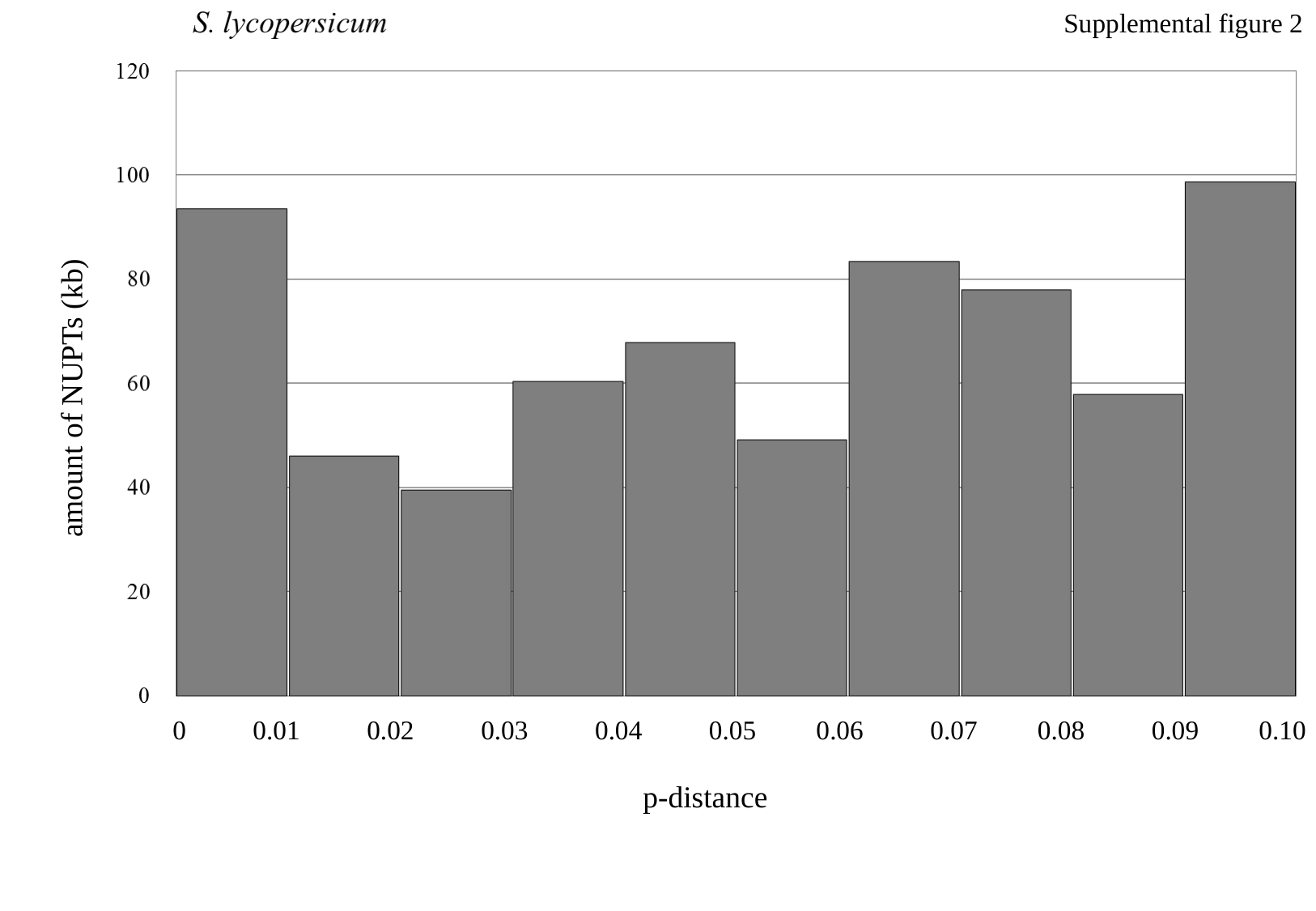

Supplemental figure 2
amount of NUPTs (kb)
 0 0.01 0.02 0.03 0.04 0.05 0.06 0.07 0.08 0.09 0.10
p-distance

## Slide 10
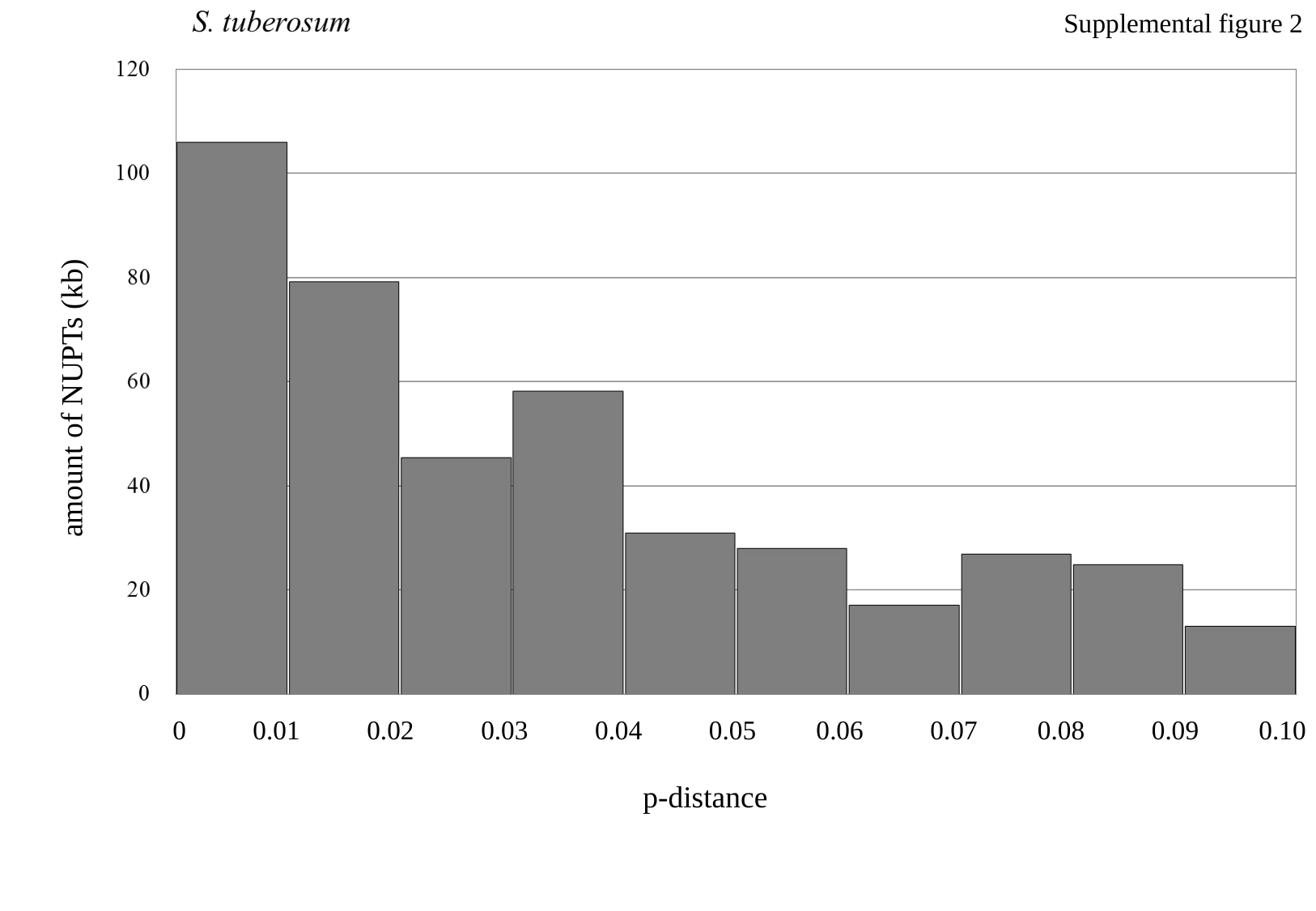

Supplemental figure 2
amount of NUPTs (kb)
 0 0.01 0.02 0.03 0.04 0.05 0.06 0.07 0.08 0.09 0.10
p-distance

## Slide 11
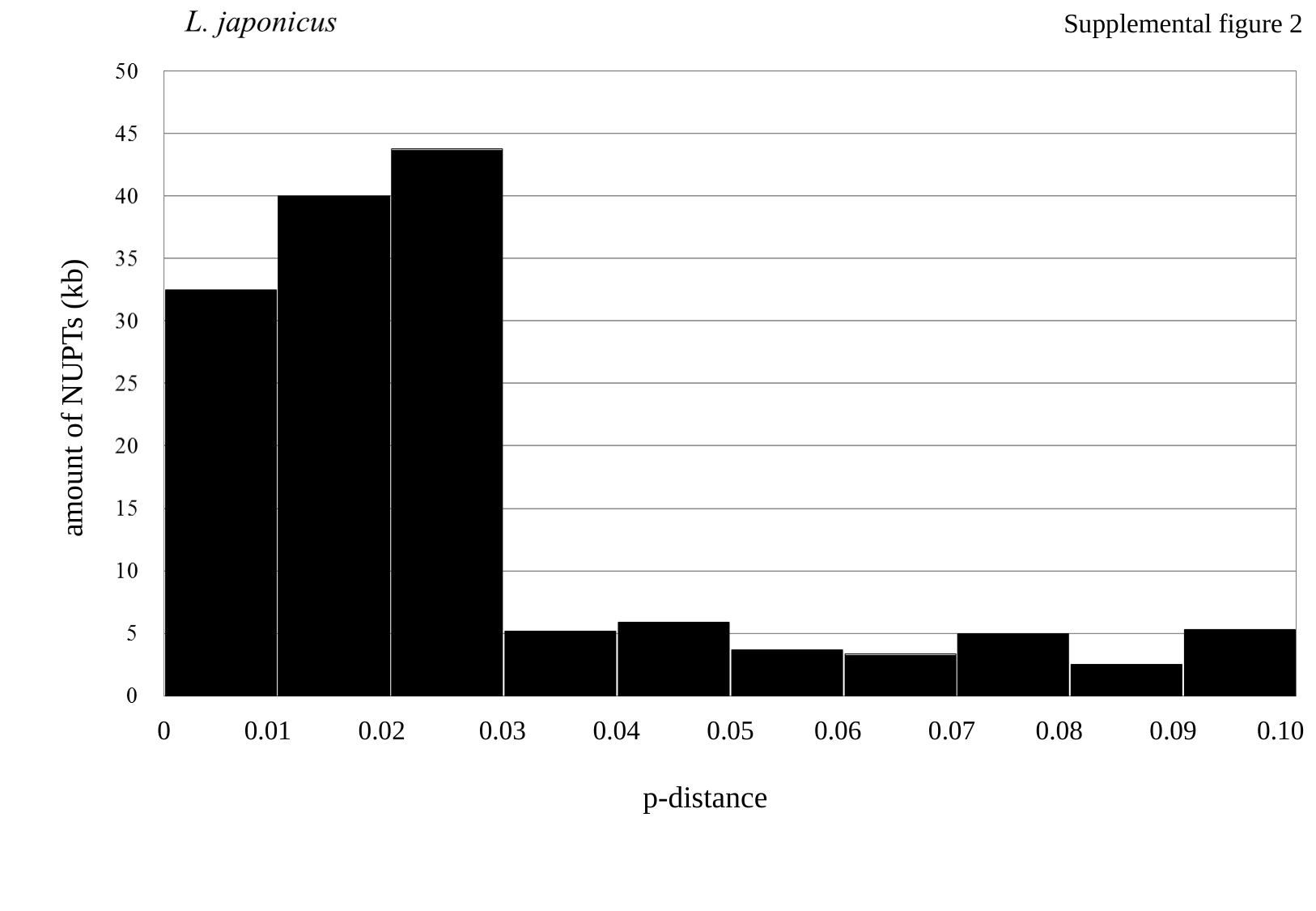

Supplemental figure 2
amount of NUPTs (kb)
 0 0.01 0.02 0.03 0.04 0.05 0.06 0.07 0.08 0.09 0.10
p-distance

## Slide 12
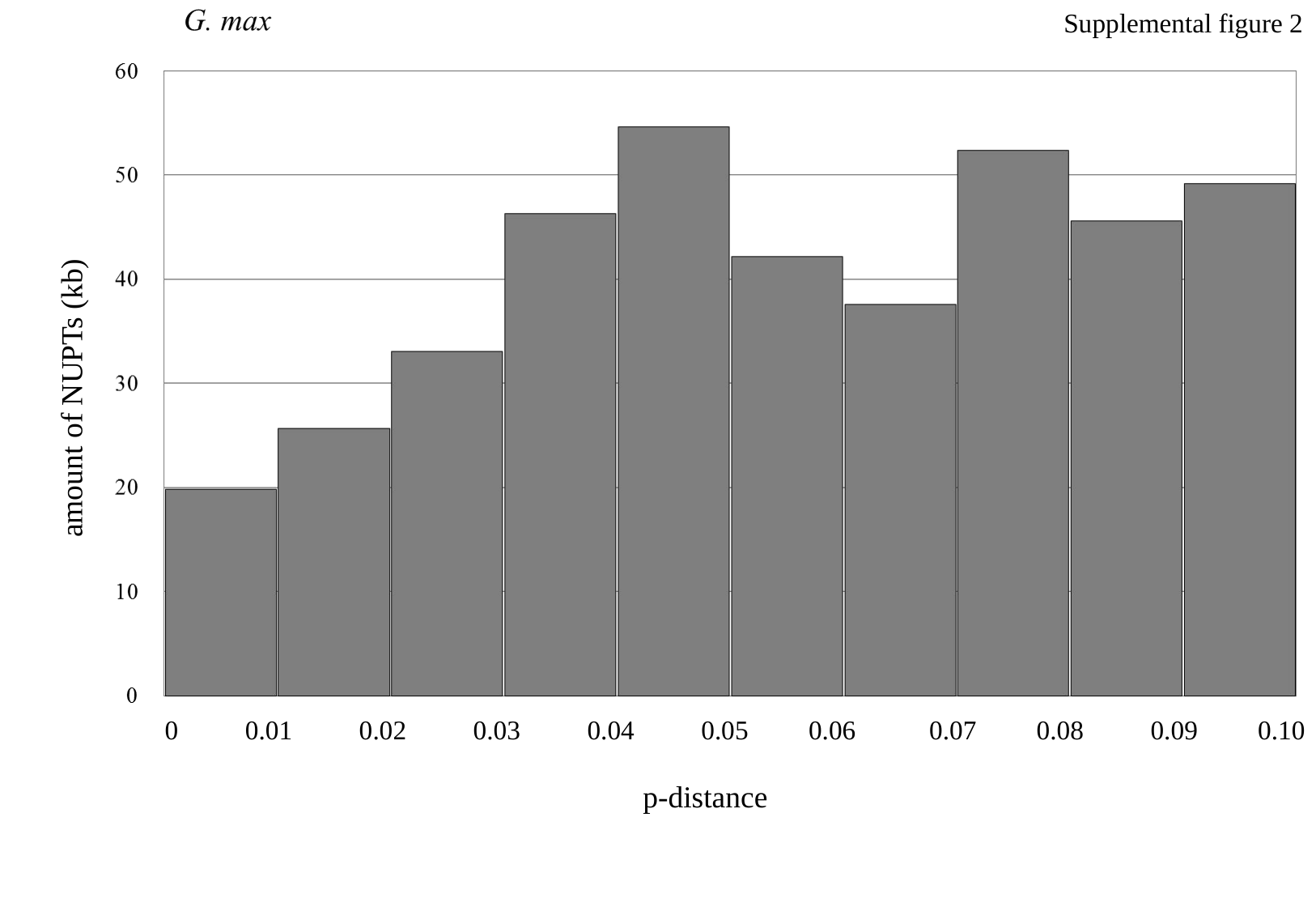

Supplemental figure 2
amount of NUPTs (kb)
 0 0.01 0.02 0.03 0.04 0.05 0.06 0.07 0.08 0.09 0.10
p-distance

## Slide 13
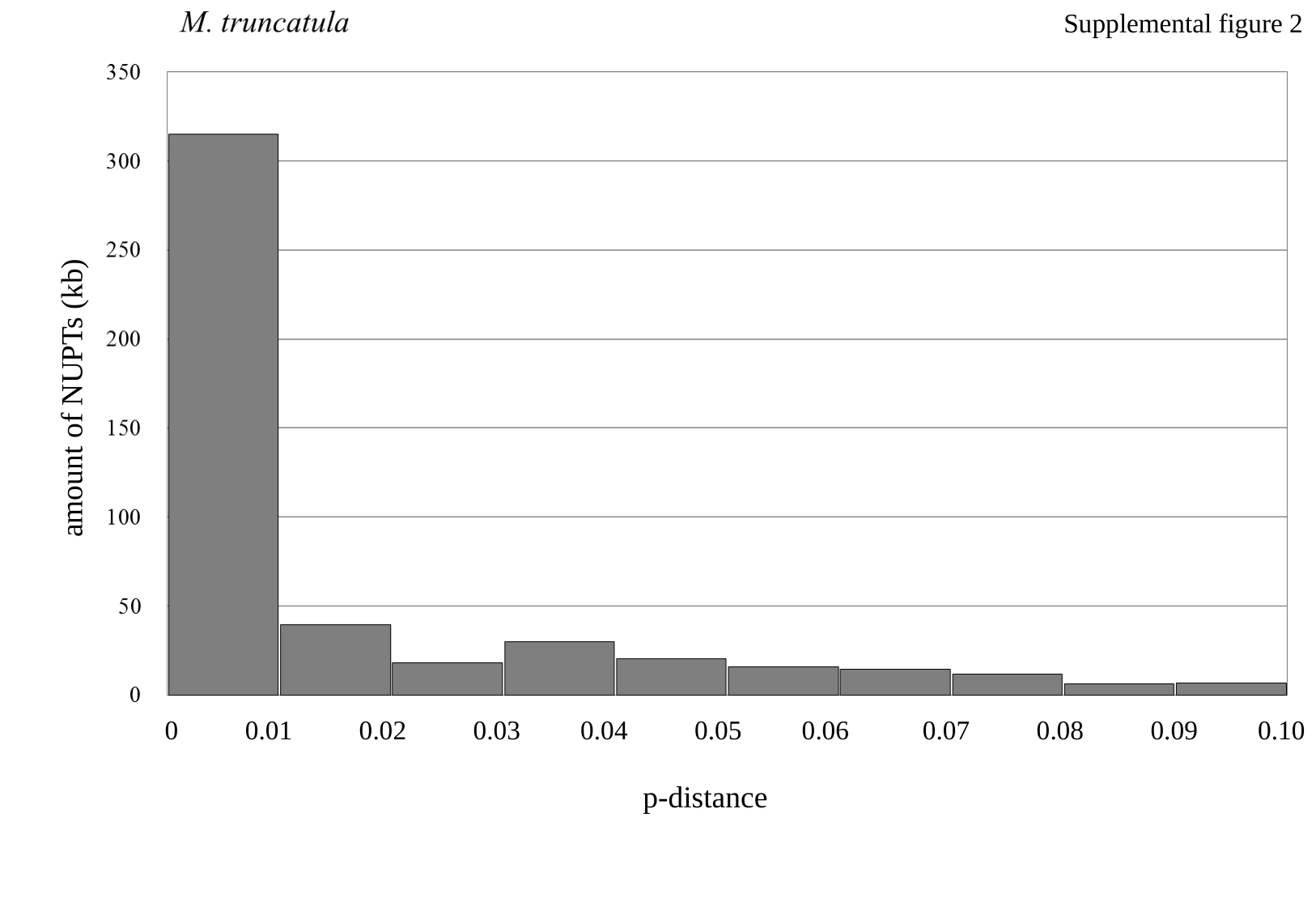

Supplemental figure 2
amount of NUPTs (kb)
 0 0.01 0.02 0.03 0.04 0.05 0.06 0.07 0.08 0.09 0.10
p-distance

## Slide 14
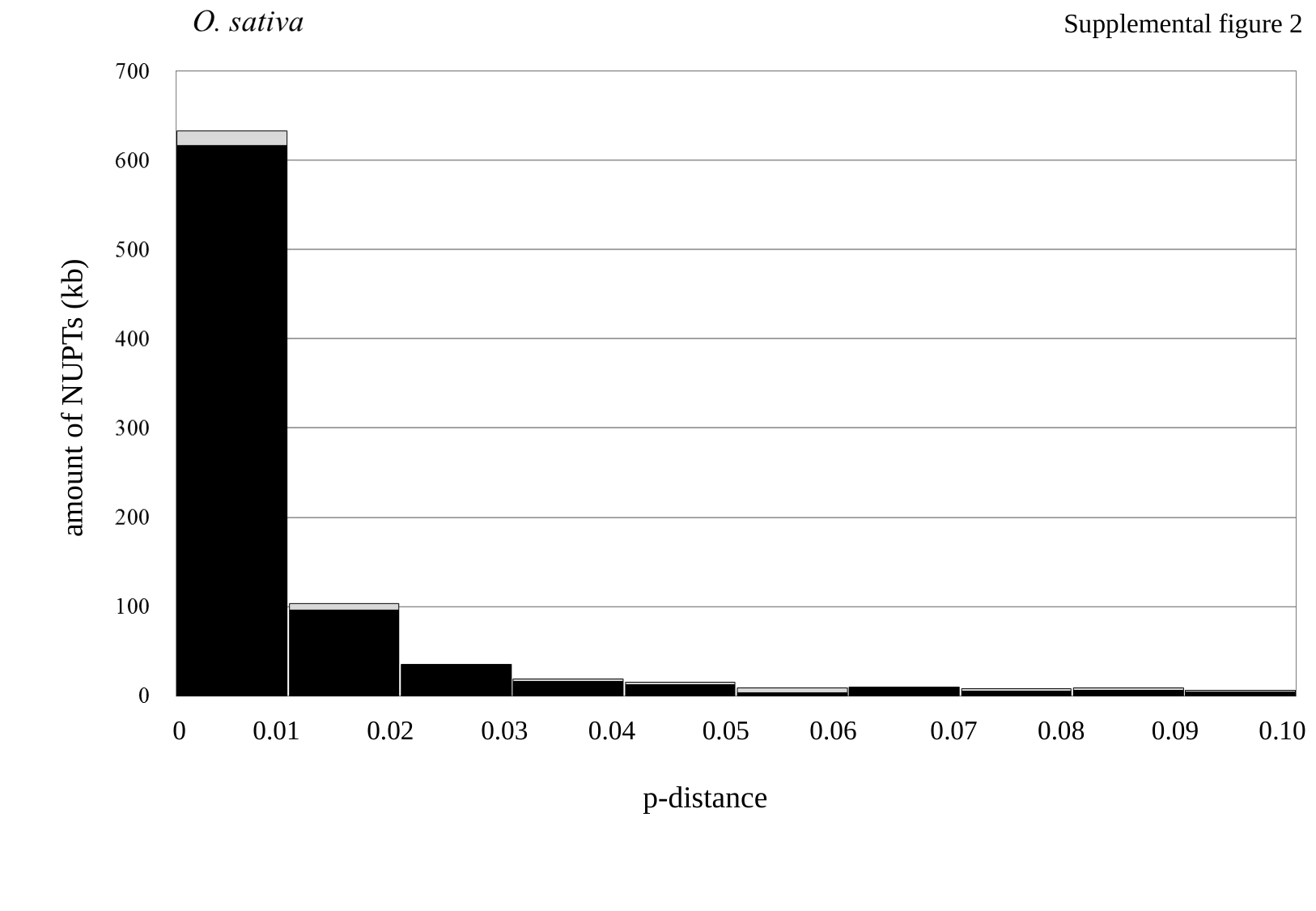

Supplemental figure 2
amount of NUPTs (kb)
 0 0.01 0.02 0.03 0.04 0.05 0.06 0.07 0.08 0.09 0.10
p-distance

## Slide 15
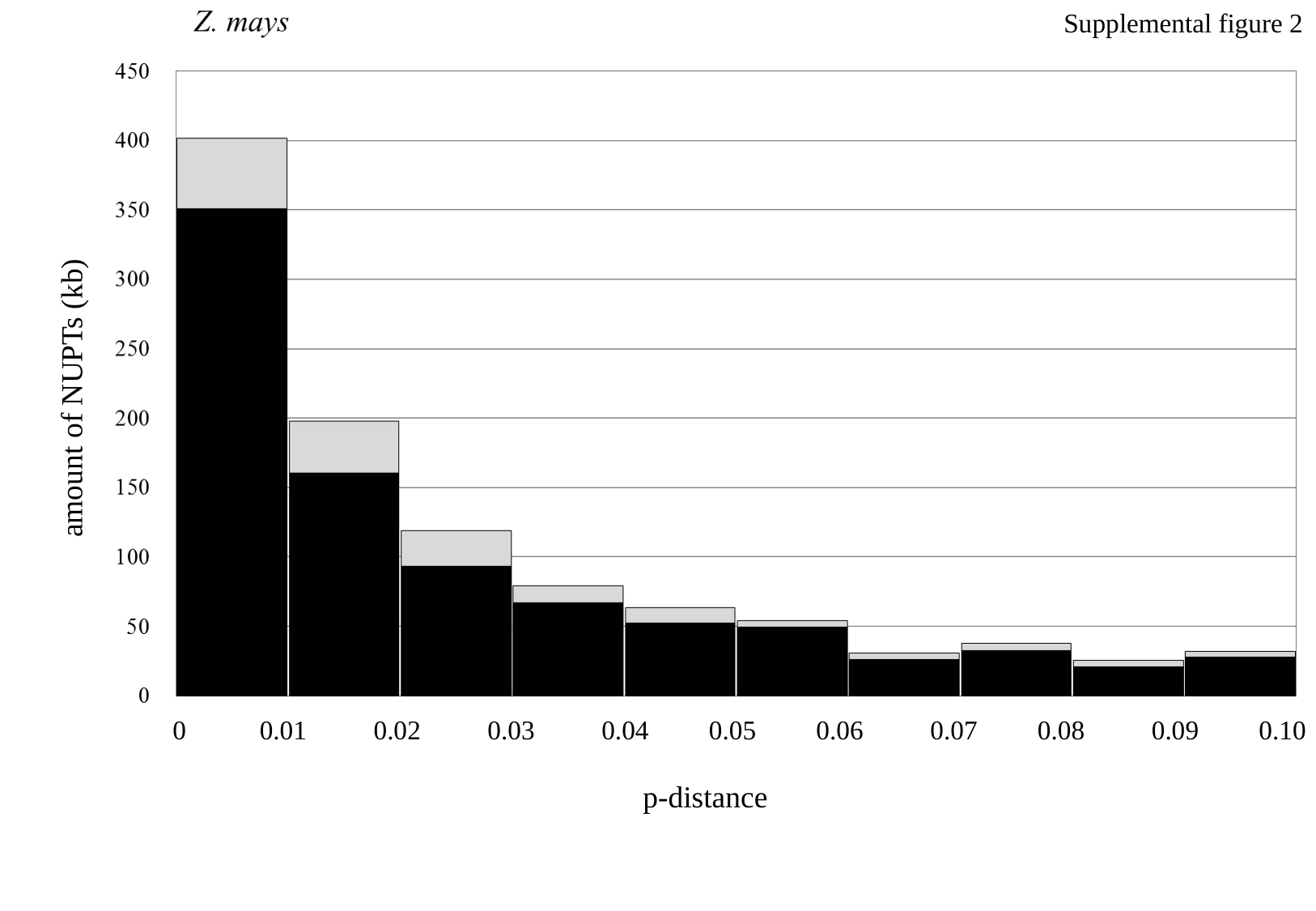

Supplemental figure 2
amount of NUPTs (kb)
 0 0.01 0.02 0.03 0.04 0.05 0.06 0.07 0.08 0.09 0.10
p-distance

## Slide 16
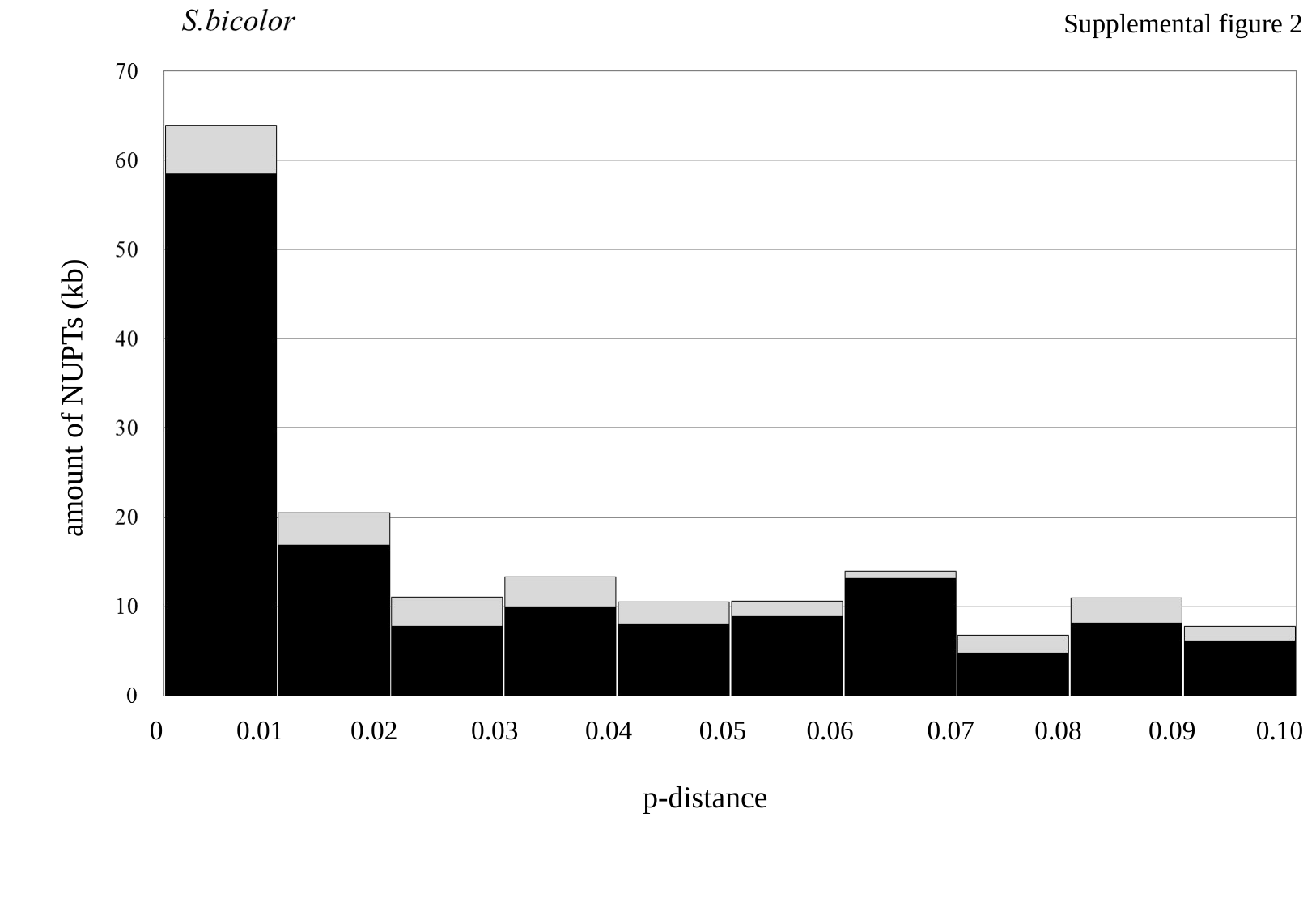

Supplemental figure 2
amount of NUPTs (kb)
 0 0.01 0.02 0.03 0.04 0.05 0.06 0.07 0.08 0.09 0.10
p-distance

## Slide 17
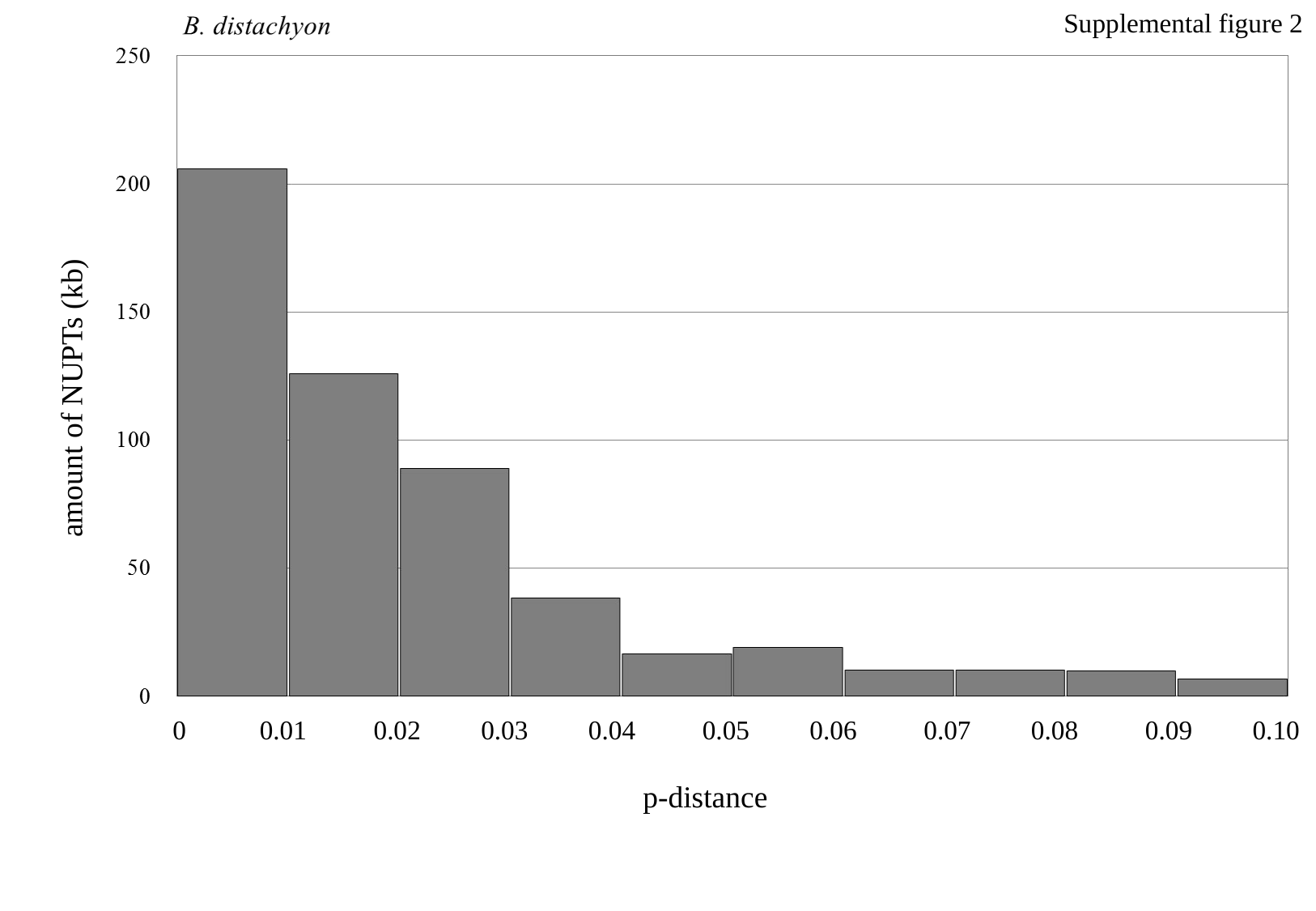

Supplemental figure 2
amount of NUPTs (kb)
 0 0.01 0.02 0.03 0.04 0.05 0.06 0.07 0.08 0.09 0.10
p-distance
