# Supplementary material for: Patterns of Genomic Integration of Nuclear Chloroplast DNA Fragments in Plant Species
Source: DNA Res. 2013 Oct 29;21(2):127–40. doi: 10.1093/dnares/dst045 (PMC3989485; doi:10.1093/dnares/dst045)
Supplement: Supplementary Data [file supp_dst045_dst045supp_fig3.ppt]

## Slide 1
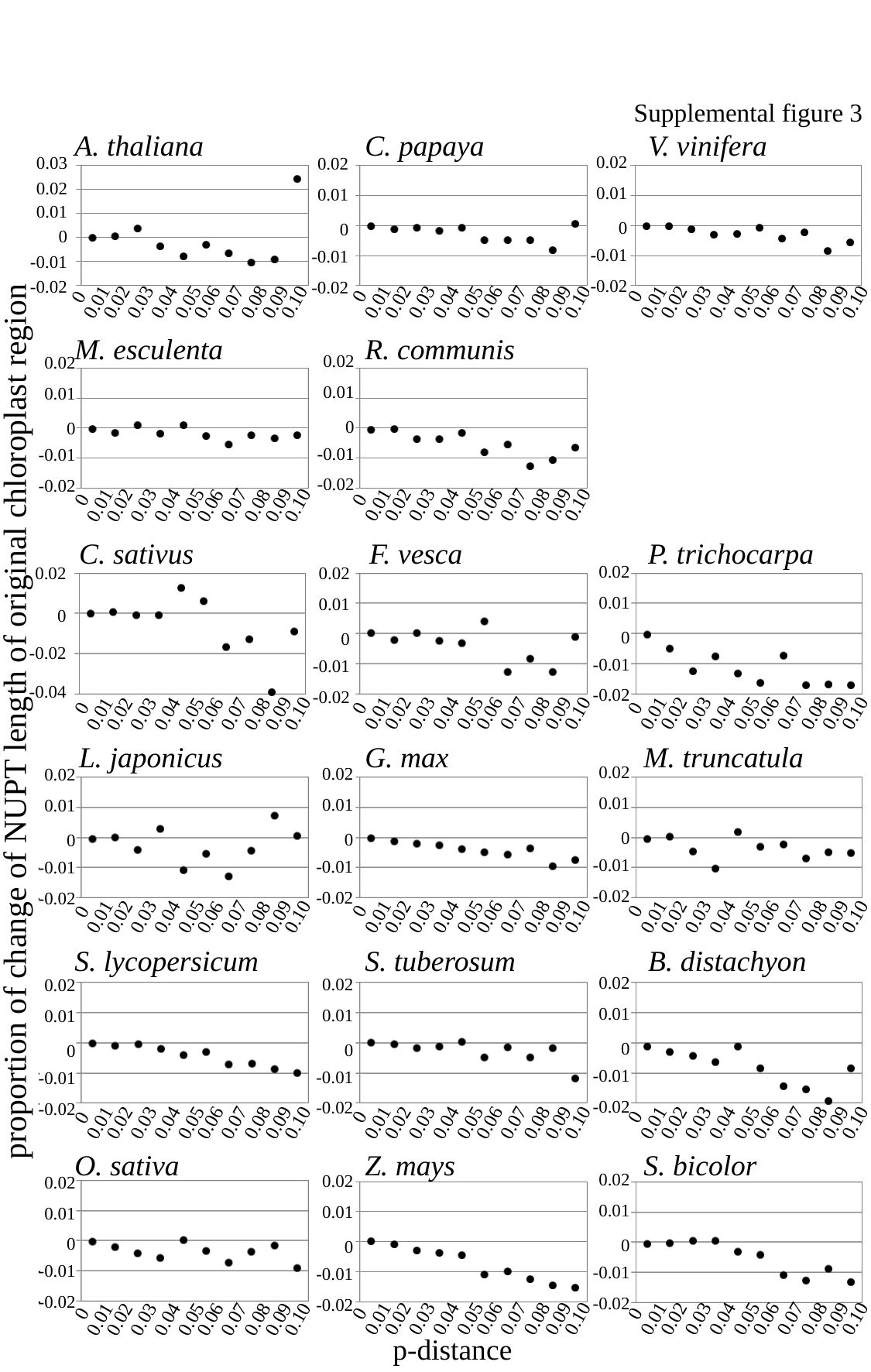

Supplemental figure 3
| A. thaliana | C. papaya | V. vinifera |
| --- | --- | --- |
| M. esculenta | R. communis | |
| C. sativus | F. vesca | P. trichocarpa |
| L. japonicus | G. max | M. truncatula |
| S. lycopersicum | S. tuberosum | B. distachyon |
| O. sativa | Z. mays | S. bicolor |
0.02
0.01
0
-0.01
-0.02
0.03
0.02
0.01
0
-0.01
-0.02
0.02
0.01
0
-0.01
-0.02
0
0.09
0.10
0.01
0.02
0.03
0.04
0.05
0.06
0.07
0.08
0
0.09
0.10
0.01
0.02
0.03
0.04
0.05
0.06
0.07
0.08
0
0.09
0.10
0.01
0.02
0.03
0.04
0.05
0.06
0.07
0.08
0.02
0.01
0
-0.01
-0.02
0.02
0.01
0
-0.01
-0.02
0
0.09
0.10
0.01
0.02
0.03
0.04
0.05
0.06
0.07
0.08
0
0.09
0.10
0.01
0.02
0.03
0.04
0.05
0.06
0.07
0.08
0.02
0.01
0
-0.01
-0.02
0.02
0
-0.02
-0.04
0.02
0.01
0
-0.01
-0.02
0
0.09
0.10
0.01
0.02
0.03
0.04
0.05
0.06
0.07
0.08
0
0.09
0.10
0.01
0.02
0.03
0.04
0.05
0.06
0.07
0.08
0
0.09
0.10
0.01
0.02
0.03
0.04
0.05
0.06
0.07
0.08
proportion of change of NUPT length of original chloroplast region
0.02
0.01
0
-0.01
-0.02
0.02
0.01
0
-0.01
-0.02
0.02
0.01
0
-0.01
-0.02
0
0.09
0.10
0.01
0.02
0.03
0.04
0.05
0.06
0.07
0.08
0
0.09
0.10
0.01
0.02
0.03
0.04
0.05
0.06
0.07
0.08
0
0.09
0.10
0.01
0.02
0.03
0.04
0.05
0.06
0.07
0.08
0.02
0.01
0
-0.01
-0.02
0.02
0.01
0
-0.01
-0.02
0.02
0.01
0
-0.01
-0.02
0
0.09
0.10
0.01
0.02
0.03
0.04
0.05
0.06
0.07
0.08
0
0.09
0.10
0.01
0.02
0.03
0.04
0.05
0.06
0.07
0.08
0
0.09
0.10
0.01
0.02
0.03
0.04
0.05
0.06
0.07
0.08
0.02
0.01
0
-0.01
-0.02
0.02
0.01
0
-0.01
-0.02
0.02
0.01
0
-0.01
-0.02
0
0.09
0.10
0.01
0.02
0.03
0.04
0.05
0.06
0.07
0.08
0
0.09
0.10
0.01
0.02
0.03
0.04
0.05
0.06
0.07
0.08
0
0.09
0.10
0.01
0.02
0.03
0.04
0.05
0.06
0.07
0.08
p-distance
