# Supplementary material for: Patterns of Genomic Integration of Nuclear Chloroplast DNA Fragments in Plant Species
Source: DNA Res. 2013 Oct 29;21(2):127–40. doi: 10.1093/dnares/dst045 (PMC3989485; doi:10.1093/dnares/dst045)
Supplement: Supplementary Data [file supp_dst045_dst045supp_tables.doc]

Supplemental Table S1. Data resource of genome sequences used in this study

| Taxon | Source | NCBI BRAST database | Accession number /  bulk data file name | Data collection date |
| --- | --- | --- | --- | --- |
| dicot: |  |  |  |  |
| *Arabidopsis thaliana* | GenBank | NCBI Chromosome Sequences | NC_003070.9, NC_003071.7, NC_003074.8, NC_003075.7, NC_003076.8 | April 10, 2012 |
| *Carica papaya* | Phytozome |  | Cpapaya_113 | May 20, 2012 |
| *Vitis vinifera* | Phytozome |  | Vvinifera_145 | May 22, 2012 |
| *Lotus japonicus* | Kazusa DNA Research Institute  http://www.kazusa.or.jp/lotus/ |  | lotus_r2.5 | October 9, 2012 |
| *Medicago truncatula* | GenBank | Medicago truncatula genomic (reference only) |  | June 13, 2012 |
| *Glycine max* | GenBank | Glycine max assembly V1.0 [GCF_000004515.1] scaffolds (reference assembly in build 1.1) |  | June 13, 2012 |
| *Manihot esculenta* | Phytozome |  | Mesculenta_147 | July 13, 2012 |
| *Ricinus communis* | GenBank | NCBI Chromosome Sequences |  | June 8, 2012 |
| *Populus trichocarpa* | GenBank | Populus trichocarpa genomic (reference only) |  | June 8, 2012 |
| *Cucumis sativus* | GenBank | NCBI Chromosome Sequences |  | March 21, 2013 |
| *Fragaria vesca* | Genome Database for Rosaceae.  http://www.rosaceae.org/ |  | Whole Genome Assemblies (v1.1) | July 13, 2012 |
| *Solanum tuberosum* | Solanaceae Genomics Resource.  http://solanaceae.plantbiology.msu.edu/ |  | PGSC_DM_v3_scaffolds | July 13, 2012 |
| *Solanum lycopersicum* | The International Tomato Genome Sequencing Consortium  http://solgenomics.net/organism/Solanum_lycopersicum/genome |  | S_lycopersicum_chromosomes.2.40 | July 13, 2012 |
| monocot: |  |  |  |  |
| *Brachypodium distachyon* | GenBank | NCBI Chromosome Sequences | NC_16131.1 - NC_16135.1 | May 9, 2013 |
| *Oryza sativa* | GenBank | NCBI Chromosome Sequences | NC_008394- NC_008405 | May 16, 2012 |
| *Sorghum bicolor* | GenBank | NCBI Chromosome Sequences | NC_012870.1- NC_012879.1  NW_002994219.1- NW_002994226.1 | April 18, 2012 |
| *Zea mays* | Phytozome | NCBI Chromosome Sequences | Zmays_181 | May 22, 2012 |

Supplemental Table S2. Source and accession number of organelle DNA sequences

Taxon Source accession number

Chloroplast Mitochondria

Dicot species

*Arabidopsis thaliana* NC_000932.1 NC_001284.2

*Carica papaya* EU431223.1 NC_012116.1

*Vitis vinifera* DQ424856.1 NC_012119.1

*Lotus japonicus* NC_002694.1 NC_016743.1

*Medicago truncatula* NC_003119.6

*Glycine max* DQ317523.1

*Manihot esculenta* NC_010433.1

*Ricinus communis* JF937588.1

*Populus trichocarpa* EF489041.1

*Cucumis sativus* AJ970307.1

*Fragaria vesca* NC_015206.1

*Solanum tuberosum* DQ386163.2

*Solanum lycopersicum* NC_007898

Monocot species

*Brachypodium distachyon* EU325680.1

*Oryza sativa* GU592207.1 NC_011033.1

*Sorghum bicolor* EF115542.1 NC_008360.1

*Zea mays* X86563.2 Phytozome (Zmays_181)

Supplemental Table S3. Distribution of NUPTs in the chloroplast genome

Mitochondria homologous vs. non-homologous region

Amount of NUPTs per 1 bp Welch Two-Sample *t*-test

Species mit-chl other regions *t* df

*Arabidopsis thaliana* 1.17665 0.09153 79.1486 * 4869.4

*Carica papaya* 1.76647 1.65680 17.2504 * 86971.8

*Vitis vinifera* 2.41813 1.87288 57.9130 * 119676.5

*Lotus japonicus* 1.35806 1.04400 34.5015 * 3746.6

*Oryza sativa* 7.07130 6.15608 61.1320 * 26078.6

*Sorghum bicolor* 1.30100 1.17125 18.6278 * 48111.7

*Zea mays* 8.27450 7.05938 66.4438 * 94931.9

*: *p* < 0.001

Supplemental Table S4. Estimation of number of NUPTs

Species chloroplast NUPTs longer than 1 kb NUPTs longer than 5 kb Number of NUPTs with p-distances <0.01

Based on >1 kb NUPTs Based on >5 kb NUPTs

genome size Number Proportion Estimated Number Proportion Estimated Observed Expected^2^ Obs./Exp. Expected^2^ Obs./Exp.

(bp) (%) transfer rate^1^ (%) transfer rate^1^

*A. thaliana* 154478 3 7.895 0.0815 1 2.632 0.0734 3 81527.3 0.000037 73377.1 0.000041

*C. papaya* 160100 49 7.993 0.0835 2 0.326 0.6134 88 83458.4 0.001054 613383.1 0.000143

*V. vinifera* 160928 48 5.333 0.1257 1 0.111 1.8104 82 125732.9 0.000652 1810440.0 0.000045

*L. japonicus* 150519 20 5.076 0.1236 5 1.269 0.1483 88 123554.5 0.000712 148261.2 0.000594

*M. truncatula* 161453 82 22.715 0.0296 22 6.094 0.0331 83 29615.7 0.002803 33116.2 0.002506

*G. max* 163161 28 1.951 0.3485 4 0.279 0.7317 72 348455.9 0.000207 731675.1 0.000098

*M. esculenta* 157033 4 2.01 0.3255 0 0.000 na 18 325524.5 0.000055 na na

*R. communis* 124033 47 7.437 0.0695 4 0.633 0.2450 63 69491.0 0.000907 244965.2 0.000257

*P. trichocarpa* 152218 114 38.908 0.0163 1 0.341 0.5575 114 16301.1 0.006993 557498.4 0.000204

*C. sativus* 155293 5 2.959 0.2187 0 0.000 na 41 218673.3 0.000187 na na

*F. vesca* 155691 6 2.752 0.2357 0 0.000 na 39 235724.0 0.000165 na na

*S. tuberosum* 155296 101 17.94 0.0361 10 1.776 0.1093 96 36068.4 0.002662 109289.6 0.000878

*S. lycopersicum* 155461 112 7.403 0.0883 12 0.793 0.2450 180 88298.0 0.002039 245013.0 0.000735

*B. distachyon* 135199 80 9.27 0.0608 17 1.970 0.0858 243 60769.1 0.003999 85791.7 0.002832

*O. sativa* 134551 116 18.985 0.0295 33 5.401 0.0311 176 29530.1 0.00596 31140.4 0.005652

*S. bicolor* 140754 25 4.854 0.1208 2 0.388 0.4531 138 120823.0 0.001142 453051.9 0.000305

*Z. mays* 140384 200 13.708 0.0427 32 2.193 0.0800 330 42671.0 0.007734 80007.9 0.004125

^1^: Transfer rate per generation was estimated by using actual transfer rate (1/16,000), proportion of NUPTs longer than 1 kb or 5 kb, and proportion of 1.5-kb region in each chloroplast genome (chloroplast genome size/1,500 bp).

^2^: Expected number of NUPTs with p-distances less than 0.1 was estimated as multiple estimated transfer rate per generation by number of generation (0.01/10^-8^).
